# Siponimod inhibits disease-associated microglia-T cell interactions in chronic experimental autoimmune encephalomyelitis

**DOI:** 10.1186/s40478-025-02136-3

**Published:** 2025-12-01

**Authors:** Leila Husseini, Anastasia Geladaris, Marlene C. J. Steinleitner, Darius Häusler, Martin S. Weber

**Affiliations:** 1 Department of Neurology, University Medical Center Göttingen, Göttingen, Germany; 2 Institute of Neuropathology, University Medical Center Göttingen, Göttingen, Germany; 3 Fraunhofer Institute for Translational Medicine and Pharmacology ITMP, Translational Neuroinflammation and Automated Microscopy TNM, Göttingen, Germany

**Keywords:** Multiple sclerosis, Neurodegeneration, Progression, Microglia, Siponimod

## Abstract

Chronically inflamed, reactive microglia represent a prominent feature of secondary progressive multiple sclerosis (SPMS). Especially their interplay with encephalitogenic T cells promotes neuroaxonal damage associated with disease progression. In our study, we aimed to explore the potential of siponimod, a sphingosine-1-phosphate modulator approved for the treatment of active SPMS, to inhibit disease-associated T cell-microglia interactions using a chronic murine experimental autoimmune encephalomyelitis (EAE) model of MS. We found that therapeutic siponimod treatment of chronic EAE improved clinical severity accompanied by reduced demyelination and neuroaxonal damage, diminished CNS T cell infiltration and altered proinflammatory microglia responses. This effect was partly attributed to a direct effect on microglia, as siponimod pretreatment inhibited interferon-γ-elicited responses of primary mouse microglia in vitro and limited their ability to induce T cell activation and proliferation in T cell-microglia co-cultures. Additionally, we observed reduced peripheral T cell numbers in our EAE model, with a pronounced shift to immunosenescent and regulatory T cell subsets, a pattern which we similarly detected in a cohort of SPMS patients following siponimod treatment. These findings indicate that siponimod dampens compartmentalized CNS inflammation by disrupting detrimental interactions between T cells and microglia through a dual central and peripheral mechanism of action.

**Supplementary Information:**

The online version contains supplementary material available at 10.1186/s40478-025-02136-3.

## Introduction

Multiple sclerosis (MS), an inflammatory and demyelinating disease of the central nervous system (CNS), is one of the most common causes of disability in young adults. Physical impairment can arise from acute relapses, or alternatively from steady chronic disease progression. While acute MS flares develop upon CNS infiltration of immune cells forming an acute lesion, chronic progression is thought to arise from a self-perpetuating inflammatory circuit between CNS-invading immune cells and CNS-resident cells, in which reactive microglia play presumably a central role [1]. In the chronic course of MS, this compartmentalized inflammation is increasingly independent of de novo infiltration of immune cells. Accordingly, all existing MS therapies exclusively targeting the activity of the peripheral immune system essentially failed to control chronic progression of MS [2].

Siponimod, an oral sphingosine-1-phosphate (S1P) receptor modulator, was developed on the basis of the first-generation S1P agonist fingolimod, a less selective functional S1P receptor antagonist approved for the treatment of relapsing–remitting (RR)MS [3, 4], but not for progressive MS [5]. Siponimod became the first oral drug approved for active secondary progressive (SP)MS, based on the results of the pivotal EXPAND trial, which demonstrated a significant reduction in the risk of confirmed disability progression and brain atrophy [6]. Siponimod preferably binds to two of the five S1P receptor subtypes, S1P receptor 1 and 5. Similar to fingolimod, a key molecular mechanism of action of siponimod is the internalization and degradation of S1P1 receptors on lymphocytes, thereby preventing the egress of T and B lymphocytes from secondary lymphoid organs and thus reducing their infiltration into the CNS [7]. Of importance, siponimod has been shown to reach the CNS compartment [8, 9], and S1P receptors 1 and 5 are expressed to a varying degree on CNS-resident cells including microglia [10]. These represent two key prerequisites for postulating a CNS-intrinsic effect of siponimod. However, the extent to which the reported positive effects of siponimod in patients with active SPMS are mediated by direct or indirect modulation of glial cells remains unknown.

To address this key question, we designed an experimental study to investigate whether siponimod treatment abrogates disease-associated microglia responses in vivo and to what extent such an effect can ameliorate chronic CNS inflammation in an experimental model of MS. To this end, therapeutic siponimod treatment was initiated in a chronic experimental autoimmune encephalomyelitis (EAE) model of MS. In addition, in vitro experiments were carried out to investigate whether siponimod is able to exert direct effects on microglia and to what extent this affects their functional properties. Furthermore, peripheral blood mononuclear cells (PBMCs) from 15 RRMS patients treated with fingolimod and 20 SPMS patients treated with siponimod were analyzed and the data were compared to age- and sex-matched untreated and healthy controls. To complement the analysis, an additional longitudinal evaluation was conducted in 13 of 20 siponimod treated SPMS patients.

## Results

### Therapeutic siponimod treatment of chronic experimental autoimmune encephalomyelitis (EAE) leads to ameliorated clinical disease with reduction of spinal cord demyelination and neuroaxonal damage

**Fig. 1:**
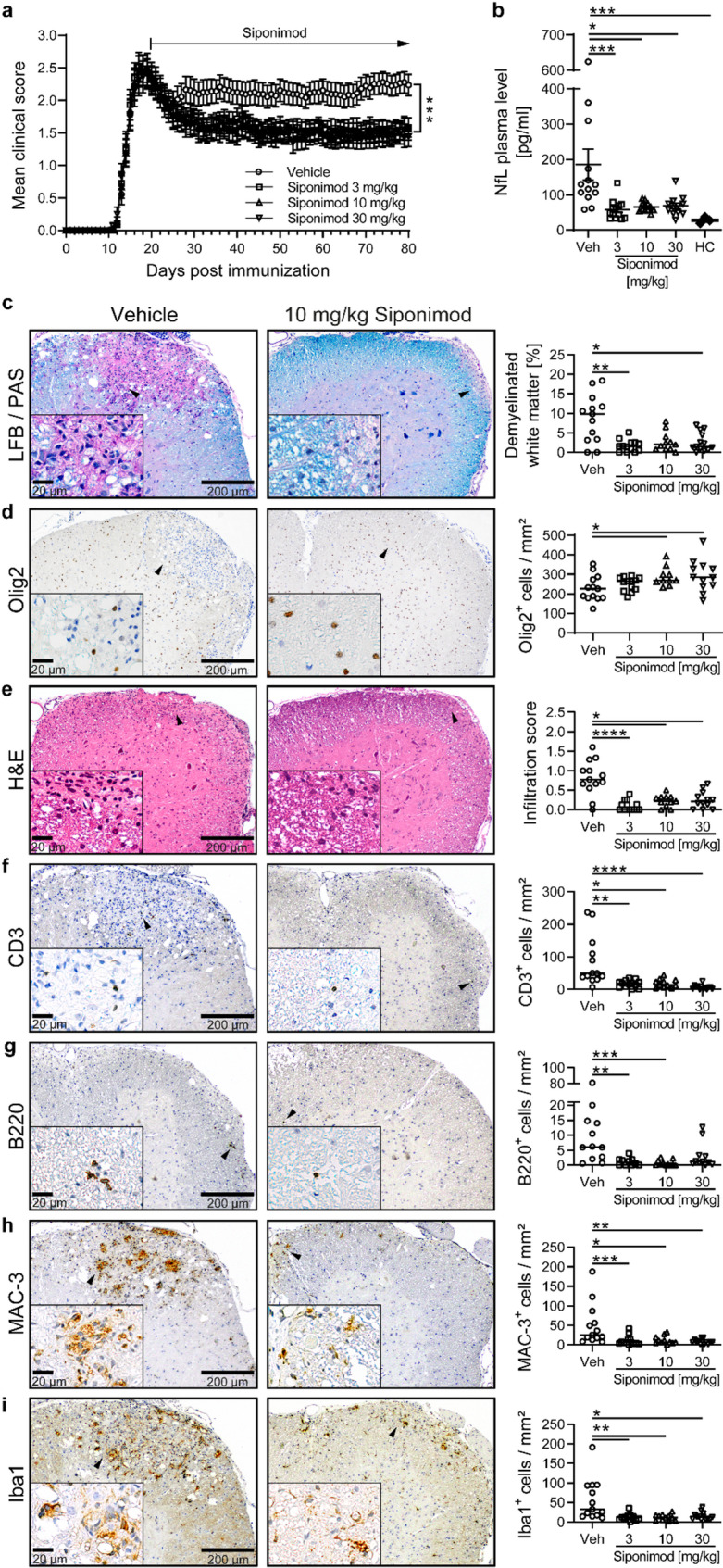
Therapeutic siponimod treatment of chronic EAE leads to clinical disease amelioration associated with reduced demyelination and neuroaxonal damage. EAE was induced by immunization with MOG peptide 35–55. Therapeutic treatment with food pellets loaded with siponimod at three different concentrations of 3, 10 or 30 mg per kg of food was initiated 20 days post immunization and maintained for at least 60 days. EDTA blood samples for measurements of plasma NfL levels and spinal cords for histological analysis were collected between day 80 and 92 post immunization. **a** Mean group EAE severity is given as mean clinical score ± SEM; pooled plots of three independent experiments; n = 34–36 per treatment group; asterisks indicate the statistical difference between vehicle (Veh) and 30 mg siponimod/kg. **b** Plasma NfL concentrationsmean ± SEM; Veh, 3, 10 and 30 mg siponimod/kg: n = 12–13; healthy controls (HC): n = 3. **c** Myelinated and demyelinated spinal cord white matter areas were assessed by LFB/PAS staining (left: representative sections; black arrowheads indicate area for image at higher magnification). The percentage of demyelinated white matter was calculated relative to the whole white matter area (right: data are shown as median; n = 11–13). **d** Oligodendrocyte transcription factor 2 (Olig2), number of cells/mm2 per group (left: representative sections; black arrowheads indicate area for image at higher magnification); (right: quantitative comparison of groups given as median; n = 10–13). **e** Overall spinal cord inflammation was evaluated by haematoxylin and eosin staining and assessed on a scale from 0 to 3 as follows: 0 = no infiltration; 1 = minor infiltration; 2 = moderate infiltration; 3 = pronounced infiltration (left: representative sections; black arrowheads indicate area for image at higher magnification); (right: inflammatory scores are depicted as median; n = 11–13). **f–i** Cellular CNS infiltration was assessed by immunohistochemical staining for **f** CD3, **g** B220, **h** MAC-3 and **i** Iba1 (left: representative sections; black arrowheads indicate area for image at higher magnification); (right: quantitative comparison of groups given as median; n = 10–13). Asterisks indicate significant difference calculated (**a–c, e–i**) using one-way analysis of variance Kruskal–Wallis test corrected by Dunn’s multiple comparison and **d** using one-way analysis of variance corrected by Holm Sidak (*P ≤ 0.05, **P ≤ 0.01, ***P ≤ 0.001, ****P ≤ 0.0001)

Compartmentalized, smoldering CNS inflammation with chronic active inflammatory demyelinating lesions (CALs) is linked to secondary progression in MS [11, 12]. CALs and their magnetic resonance imaging (MRI) correlate, paramagnetic ring lesions (PRLs), are characterized by a microglia-rich, perilesional rim as zone of ongoing, slowly expanding inflammation. The aim of our study was to investigate the effect of siponimod treatment on disease-associated microglial responses to neuroinflammation as potential factors in the pathogenesis of secondary progression in MS. To address this question, we started our investigation by utilizing a model of chronic experimental acute encephalomyelitis (EAE) with a therapeutic siponimod treatment approach. For this, mice were immunized with MOG peptide 35–55 and siponimod treatment was started 20 days post immunization (dpi) after the peak of clinical disease was already reached and was maintained for a period of at least 60 days. Siponimod was orally administered via food pellets loaded with three different siponimod concentrations (3, 10 or 30 mg siponimod/kg of food pellets), dosages were chosen based on a previous study where consistent penetration/distribution of siponimod was demonstrated across species, which is suggestive of a high translational potential to human [9]. All siponimod treatment groups showed a sustained amelioration of mean clinical severity compared to vehicle-treated mice (Fig. 1a). While mean siponimod plasma levels were distinct across all three siponimod treatment groups and correlated with siponimod doses, no dosage-dependent differences of treatment effects could be observed (Supplementary Fig. 1a). To assess neuroaxonal damage, plasma neurofilament light (NfL) levels were measured, revealing elevated mean NfL concentration in untreated EAE mice compared to healthy litter mates (Fig. 1b). Moreover, the improved clinical outcome after at least 60 days of siponimod treatment was associated with a significant decrease of mean plasma NfL levels of mice treated with siponimod when compared to the vehicle group (Fig. 1b). Histological analysis of spinal cords isolated after at least 60 days of siponimod treatment showed a marked decrease of demyelinated spinal cord white matter areas in mice treated with siponimod, when compared to vehicle (Fig. 1c). Areas of demyelinated spinal cord white matter correlated with the clinical outcome of siponimod treatment groups. Likewise, no significant differences between the three distinct siponimod dosage groups were observed. We assessed the number of Oligodendrocyte transcription factor 2 (Olig2)-positive cells, commonly used as pan-oligodendrocytic marker, to further elucidate the mechanism of action of improved remyelination under siponimod treatment in this animal model (Fig. 1d). We could show that siponimod treatment of chronic EAE significantly increased Olig2-positive cell numbers in spinal cord white matter.

### Ameliorated chronic EAE under siponimod treatment is associated with a decrease of CNS T cell infiltration and Iba1-positive myeloid cells

In an additional histological analysis of neighboring spinal cord sections, we assessed the overall CNS immune cell infiltration in haematoxylin and eosin stainings using a semi-quantitative inflammatory score and quantified the number of T cells, B cells and macrophages per spinal cord white matter area in immunostainings for the corresponding lineage markers CD3, B220 and MAC-3 (Fig. 1e–h). In the vehicle treated group a moderate immune cell infiltration with a significant presence of T cells was observed, while in all siponimod treated groups a reduced infiltration of lymphocytes and MAC-3-positive cells into the spinal cord was assessed. Furthermore, the number of Iba1-positive macrophages/microglia in spinal cord sections was significantly reduced under siponimod treatment compared to the vehicle-treated group with no significant difference between the three distinct siponimod dosage groups (Fig. 1i).

### Siponimod treatment of chronic EAE alters reactive microglia states linked to neuroinflammation

**Fig. 2:**
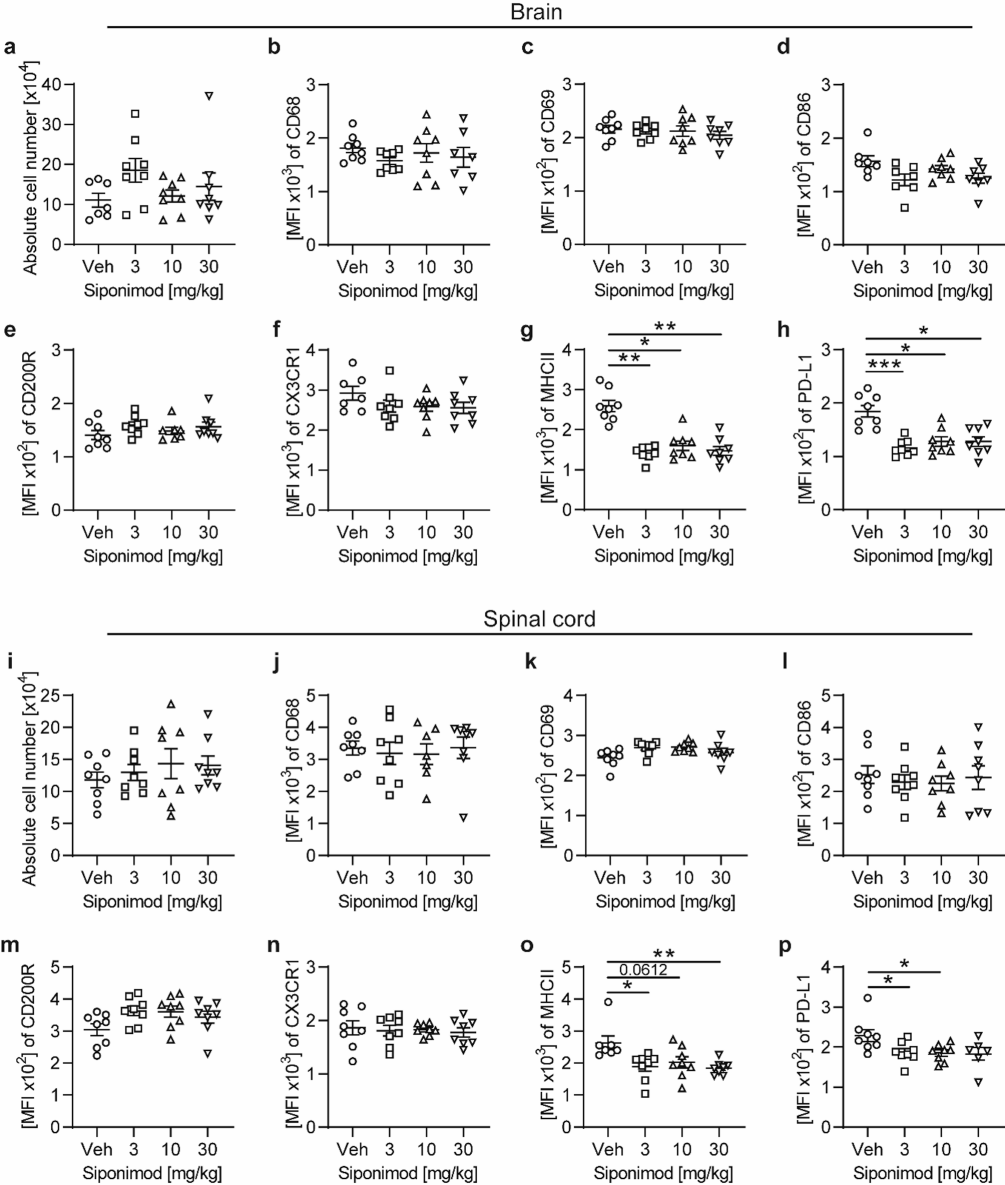
Siponimod treatment diminishes reactive microglia states in chronic EAE. EAE was induced by immunization with MOG peptide 35–55. Therapeutic treatment with food pellets loaded with siponimod at three different concentrations of 3, 10 or 30 mg per kg of food was initiated 20 days post immunization and maintained for at least 60 days. Microglia were isolated from (**a–h**) brain and (**i–p**) spinal cord and changes in expression of disease-associated markers were analyzed by flow cytometry and are shown as mean fluorescence intensity (MFI); n = 8. Mean ± standard error of the mean is indicated in all graphs. Data sets are representative of two independent experiments. Asterisks indicate significant differences calculated using one-way analysis of variance Kruskal–Wallis test corrected by Dunn’s multiple comparison (*P ≤ 0.05, **P ≤ 0.01, ***P ≤ 0.001, ****P ≤ 0.0001)

The role of reactive microglia in the context of neurodegeneration in demyelinating inflammatory CNS disorders is multifaceted and remains poorly understood, as both beneficial and detrimental processes can be attributed to microglia in the vicinity of neuroaxonal damage [13]. To therefore assess the impact of siponimod treatment on putative subpopulations of microglia, we used the same chronic EAE model and siponimod treatment plan (Fig. 2). The absolute cell numbers of microglia isolated from the brain and spinal cord did not differ between vehicle and the three siponimod treatment groups (Fig. 2a, i). Furthermore, the expression of markers linked to microglia responding to inflammatory stimuli was overall relatively low in this chronic phase of EAE. However, under siponimod treatment, a decrease in the expression of PD-L1, a marker associated with anti-inflammatory properties of myeloid cells, was observed in brain and spinal cord (Fig. 2h, p). Most prominent, therapeutic siponimod treatment across all three treatment doses induced a downregulation of microglial MHC class II expression, a marker associated with antigen-presentation, both in the spinal cord as predilection site for demyelinating inflammatory lesions in this EAE model as well as in the brain, which is typically devoid of lesions (Fig. 2g, o). Of note, other investigated markers on microglia did not change upon siponimod treatment compared to vehicle group.

### Reduced frequencies and altered phenotype of CNS infiltrating T cells under siponimod treatment of chronic EAE

**Fig. 3:**
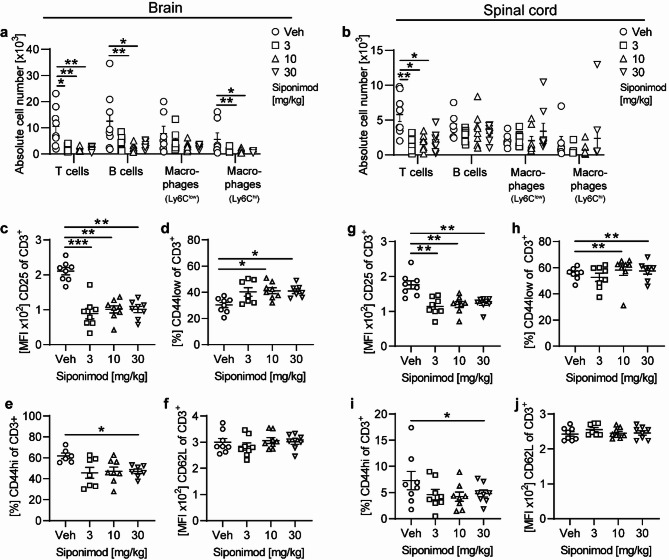
Treatment with siponimod reduces immune cell infiltration of the CNS and changes the phenotype of CNS T cell infiltrates. EAE was induced by immunization with MOG peptide 35–55. Therapeutic treatment with food pellets loaded with siponimod at three different concentrations of 3, 10 or 30 mg per kg of food was initiated 20 days post immunization and maintained for at least 60 days. Lymphocytes were isolated from (**a**), (**c**–**f**) brain and (**b**), (**g–j**) spinal cord. **a, b** Composition of brain and spinal cord-infiltrating cells (T cells: CD45+CD11b-CD3+, B cells: CD45+CD11b-CD19+; macrophages: CD11b + CD45^hi^Ly6C^low^, macrophages: CD11b+CD45^hi^Ly6C^hi^) were analyzed by flow cytometry. **c–j** Changes in expression of markers involved in activation of CD3+ T cells were analyzed by flow cytometry and are shown as mean fluorescence intensity (MFI); n = 8. Mean ± standard error of the mean is indicated in all graphs. Data sets are representative of two independent experiments. Asterisks indicate significant differences calculated using one-way analysis of variance Kruskal–Wallis test corrected by Dunn’s multiple comparison (*P ≤ 0.05, **P ≤ 0.01, ***P ≤ 0.001, ****P ≤ 0.0001)

As the microglia phenotypes we observed in EAE treated with siponimod may be influenced by the frequencies and phenotypes of adjacent CNS infiltrating immune cells, we also characterized the population of CD45^hi^ cells obtained from mouse brain and spinal cord tissue (Fig. 3). Similar to our immunohistological findings, siponimod treatment induced a marked decrease of absolute T cell numbers in both brain and spinal cord, while B lymphocytes and Ly6C^hi^ infiltrating macrophages were only significantly decreased in the brain, not the spinal cord (Fig. 3a, b).When we characterized the infiltrating T lymphocytes in brain and spinal cord, we observed a significantly reduced expression of CD25 and CD44 with a decreased proportion of CD44^hi^ T cells, both markers associated with reactivation of T cells, while the expression of the homing receptor L-selectin remained unchanged upon therapeutic siponimod treatment (Fig. 3c–j).

### Siponimod pretreatment of primary mouse microglia inhibits IFNγ-elicited microglial responses

**Fig. 4:**
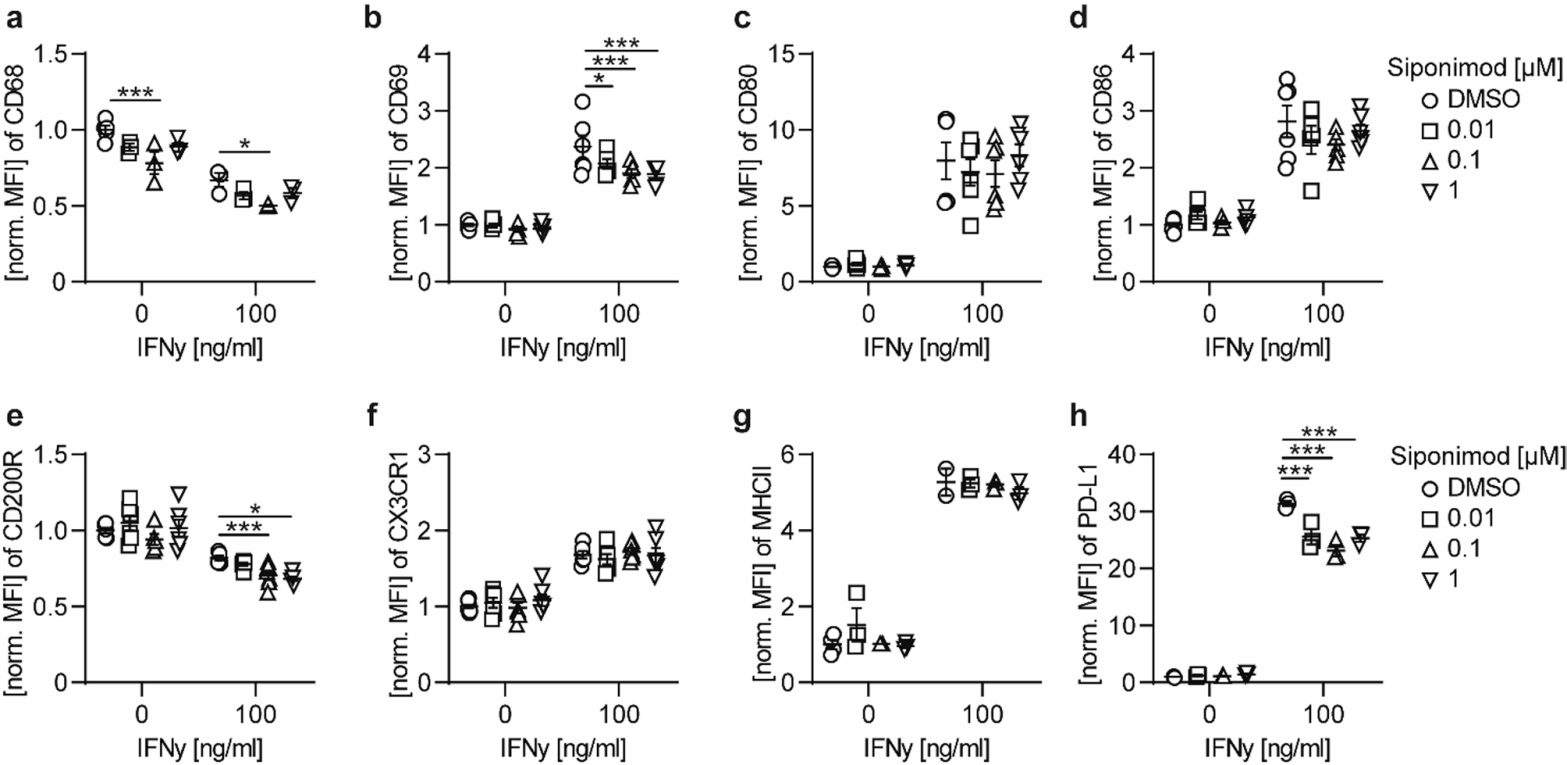
Siponimod inhibits proinflammatory T cell-microglia interactions in vitro. **a**–**h** Primary microglia were either left unstimulated or treated with indicated concentrations of siponimod or DMSO control for 24 h followed by simultaneously stimulation with 100 ng/ml IFNγ for 18 h. Changes in expression of disease-associated microglial markers were analyzed by flow cytometry and normalized to DMSO control and are shown as mean fluorescence intensity (MFI); n = 6, pooled from at least 3 independent experiments. Mean ± standard error of the mean is indicated in all graphs. Asterisks indicate significant differences calculated using one-way analysis of variance corrected by Holm-Sidak (*P ≤ 0.05, **P ≤ 0.01, ***P ≤ 0.001, ****P ≤ 0.0001)

To further elucidate the mechanism of action and the functional significance of the siponimod-induced modulation of microglia observed in chronic EAE in vivo, we next analyzed the effects of siponimod on primary murine microglia in vitro. Especially, in vitro models for neuroinflammation were utilized to address the question to which extent the siponimod effects on microglia observed in vivo are T cell-dependent or mediated by a direct modulation of microglial S1PR1. Therefore, microglia were cultured with siponimod or its solvent DMSO and challenged with three different proinflammatory stimuli, thus inducing distinct microglial phenotypes. Stimulation of microglial cultures with interferon (IFN)-γ, lipopolysaccharide (LPS) or tumor necrosis factor (TNF)-α uniformly led to an upregulation of PD-L1 and a downregulation of CD68, a marker associated with phagocytosis, and of CD200R, a marker known for the interaction of microglia with neurons (Fig. 4a–h, Supplementary Fig. 2). While both LPS- and IFNγ-stimulation of microglia induced a significant upregulation of co-stimulatory molecules CD80 and CD86 and an increased expression of the activation marker CD69, only priming of microglia cultures with IFNγ resulted in a marked increase of MHC class II expression. Pretreatment of microglia cultures with siponimod (0.01, 0.1 or 1.0 µM) induced a modulation of IFNγ-elicited microglial responses, whereas no effect on microglia reactivity to LPS and TNFα was observed (Fig. 4a–h, Supplementary Fig. 2). In particular, siponimod pretreatment suppressed the IFNγ-induced upregulation of CD69 and PD-L1 and downregulated CD200R and CD68 expression independent of IFNγ stimulation. In contrast to the siponimod treatment effect in vivo with a significant downregulation of microglial MHC class II expression in EAE mice treated with siponimod, siponimod pretreatment in vitro had no effect on IFNγ-induced microglial MHC class II upregulation. To further elucidate this inconsistency in the effect on MHC class II, we performed experiments directly assessing the effect of siponimod on IFNγ signaling cascade in primary microglia, which is known to mediate MHC class II upregulation. Therefore, we pretreated microglia with siponimod followed by IFNγ stimulation for 15 min and analyzed phosphorylation of STAT1 (pSTAT), which is activated upon binding of IFNγ to its receptor. We observed an upregulation of pSTAT1, a signaling molecule downstream of the IFNγ receptor, after stimulation with IFNγ for 15 min (Supplementary Fig. 2 s), which remained unchanged upon siponimod pretreatment (Supplementary Fig. 2 t). Overall, we observed in vitro that direct effects of the selective S1PR1 and S1PR5 modulator siponimod on the phenotype of microglia cultures were rather moderate, indicating an indirect, possibly T cell-dependent mechanism of action. Moreover, consistent with previous reports [14], a transcriptional analysis of the S1PR repertoire of primary cultured murine microglia showed only a moderate S1PR1 expression with no significant S1PR5 expression, while S1PR2 represented the most abundantly expressed S1P receptor in microglia (Supplementary Fig. 2 q, r). Furthermore, we could show a downregulation of S1PR1 in microglia stimulated with IFNγ, implying a counterregulatory mechanism reducing microglial S1PR1-mediated signaling under proinflammatory conditions.

### Bidirectional crosstalk between T cells and microglia is modulated by siponimod

**Fig. 5:**
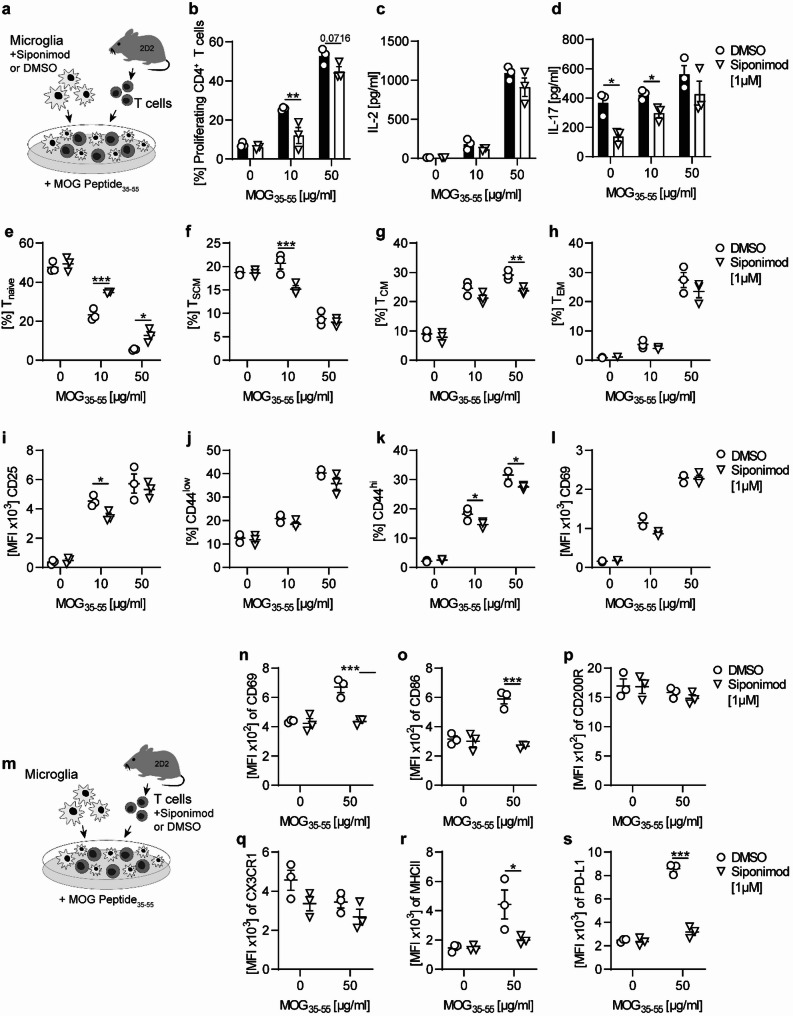
Siponimod alters T cell-microglia interactions in vitro. **a**–**l** Microglia were pre-incubated with siponimod or DMSO control and stimulated with IFNy for 18 h, followed by co-culture with MOG-specific T cells isolated from 2D2 mice in the presence of MOG peptide 35–55 for 72 h. **a** Schematic overview of experimental setting, **b** frequency of proliferating CD4+ T cells was assessed via flow cytometry using carboxyfluorescein succinimidyl ester (CFSE) dilution; n = 3; representative data from at least 3 independent experiments. **b, c** Cytokine concentrations were determined by ELISA; n = 3; representative data from at least three independent experiments. **e–h** T cell differentiation from naive to memory T cells (T_naive_: CD44-CD62L+CD95-; T_SCM_: CD44-CD62L+CD95 + ; T_CM_: CD44+CD62L+CD95+; T_EM_: CD44+CD62L-CD95+) was assessed via flow cytometry and are shown as mean fluorescence intensity, (MFI); n = 3; representative data from at least 3 independent experiments. **i–l** Changes in expression of markers associated with activation of T cells were assessed via flow cytometry; n = 3; representative data from at least 3 independent experiments. **m–s** MOG-specific T cells isolated from 2D2 mice were pre-incubated with siponimod or DMSO control for 6 h, followed by co-culture with IFNy-stimulated (18 h) microglia in the presence of MOG peptide 35–55 for 72 h. **m** Schematic overview of experimental setting. **n–s** Changes in expression of disease-associated microglial markers were analyzed by flow cytometry and are shown as mean fluorescence intensity, (MFI); n = 3, representative data from at least three independent experiments. Asterisks indicate significant differences calculated using (**a–s**) Unpaired two-tailed t-test. (*P ≤ 0.05, **P ≤ 0.01, ***P ≤ 0.001, ****P ≤ 0.0001)

Next, we aimed to investigate the impact of direct microglia modulation by siponimod on potentially detrimental, proinflammatory interactions of microglia and T cells in a T cell-microglia co-culture model for neuroinflammation simulating the interaction of reactivated myelin-specific T cells with microglia in the CNS. Hence, microglia were pretreated with 1 µM siponimod or its solvent DMSO for 18 h and subsequently co-cultured for 72 h with MOG-specific T cells isolated from transgenic 2D2 mice in the presence of MOG peptide 35–55 (Fig. 5a) and the ability of siponimod-treated microglia to activate T cells was assessed (Fig. 5b–l). When activated MOG-specific T cells engaged with microglia previously treated with siponimod, the proportion of proliferating CD4^+^ T cells was significantly reduced in comparison to the control, thus revealing a microglia-mediated inhibitory effect of siponimod treatment on T cell proliferation (Fig. 5b). Furthermore, siponimod pretreatment of microglia resulted in a modulation of T cell differentiation with a shift towards naive T cells (T_N_) and a decreased proportion of stem cell–like memory T cells (T_SCM_) and central memory T cells (T_CM_) (Fig. 5e–h). Similar to the phenotype of CNS-residing T cells observed in chronic EAE treated with therapeutic siponimod treatment in vivo (Fig. 3), siponimod pretreatment of microglia in vitro resulted in a downregulation of CD25 and a reduced proportion of CD44^hi^ T cells, while CD69 remained unchanged (Fig. 5i–l). An analysis of cytokine levels in supernatants of T cell-microglia co-cultures showed reduced concentrations of interleukin (IL)-17, a cytokine typically produced by a subset of proinflammatory T cells, when microglia was pretreated with siponimod, while IL-2 concentrations remained unchanged compared to control (Fig. 5c, d), suggesting that siponimod suppresses the capacity of microglia to (re-)activate encephalitogenic T cells. In summary, we found indications for microglia-mediated effects of siponimod on the proliferative potential and proinflammatory functions of T lymphocytes in the CNS. Since our previous data also suggest an indirect, potentially T cell-dependent mechanism of action of siponimod, we next examined the impact of siponimod-treated T cells on microglia. In this co-culture setting, siponimod-treated T cells were cultured with interferon (IFN)-γ-conditioned primary microglia (Fig. 5m–s). Upon stimulation with MOG peptide 35–55, we observed a reduction in markers associated with activation (CD69, PD-L1) and antigen presentation (CD86, MHC class II) on microglia upon co-culture with siponimod treated T cells (Fig. 5n–s). These findings support that siponimod’s mode of action involves the regulation of reciprocal interactions between T cells and microglia.

### Siponimod treatment of chronic EAE differentially alters the frequency of immune cells in blood versus lymphoid organs

**Fig. 6:**
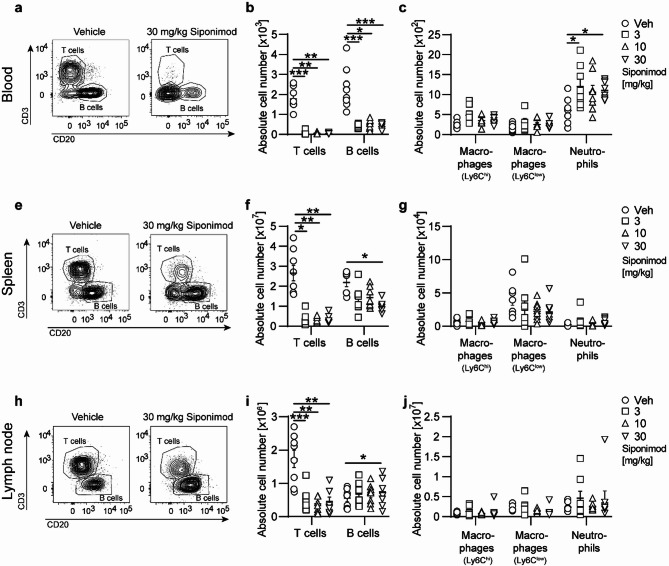
Siponimod treatment of chronic EAE differentially alters the frequency of immune cells in blood versus lymphoid organs. EAE was induced by immunization with MOG peptide 35–55. Therapeutic treatment with food pellets loaded with siponimod at three different concentrations of 3, 10 or 30 mg per kg of food was initiated 20 days post immunization and maintained for at least 60 days. Immune cells were isolated from (**a–c**) blood, **e–g** spleen,** h-j** lymph nodes. **a, e, h** Representative contour plots. **b–c, f–g, i–j** Composition of immune cells (T cells: CD45+CD11b-CD3+; B cells: CD45+CD11b-CD20+; macrophages: CD11b+CD45^hi^Ly6C^low^; macrophages: CD11b+CD45^hi^Ly6C^hi^; neutrophils: CD11b+CD45^hi^Ly6C+Ly6G+) was analyzed by flow cytometry. Absolute cell numbers are shown; n = 8. Mean ± standard error of the mean is indicated in all graphs. Data sets are representative of three independent experiments. Asterisks indicate significant differences calculated using one-way analysis of variance Kruskal–Wallis test corrected by Dunn’s multiple comparison (*P ≤ 0.05, **P ≤ 0.01, ***P ≤ 0.001, ****P ≤ 0.0001)

All S1PR modulators approved for the treatment of MS, including siponimod and fingolimod, are known to inhibit the egress of lymphocytes from lymphoid organs, especially the exit of activated T cells from lymph nodes into blood, an effect attributed to a functional antagonism on the S1PR1 of lymphocytes [15]. To further disentangle the interplay of potential central and peripheral mechanisms of action of siponimod in our chronic EAE model, we additionally characterized the impact of therapeutic siponimod treatment on the phenotype and migration of immune cells in peripheral immune cell compartments. Similar to findings in MS patients treated with S1PR modulators, total blood leukocyte counts were significantly reduced under siponimod treatment compared to vehicle, with no significant difference between the three distinct siponimod dosage groups (Supplementary Fig. 1 b). The reduced total blood leukocyte counts observed under siponimod treatment were primarily driven by a marked decline in absolute T cell numbers and, to a lesser extent, of B lymphocytes. This was accompanied by an increased relative proportion of macrophages, although their absolute numbers remained unchanged (Fig. 6a–c, Supplementary Fig. 1 c-d). Both relative proportion and absolute numbers of T lymphocytes were equally diminished in lymph nodes and spleen. In contrast, the relative proportion of B lymphocytes increased in both lymphoid organs with just a moderate reduction of absolute B cell numbers in spleen and an increased absolute B lymphocyte frequency in lymph nodes (Fig. 6e–j, Supplementary Fig. 1e-h).

### Shift to an immunosenescent, regulatory T cell phenotype in EAE treated with siponimod

**Fig. 7:**
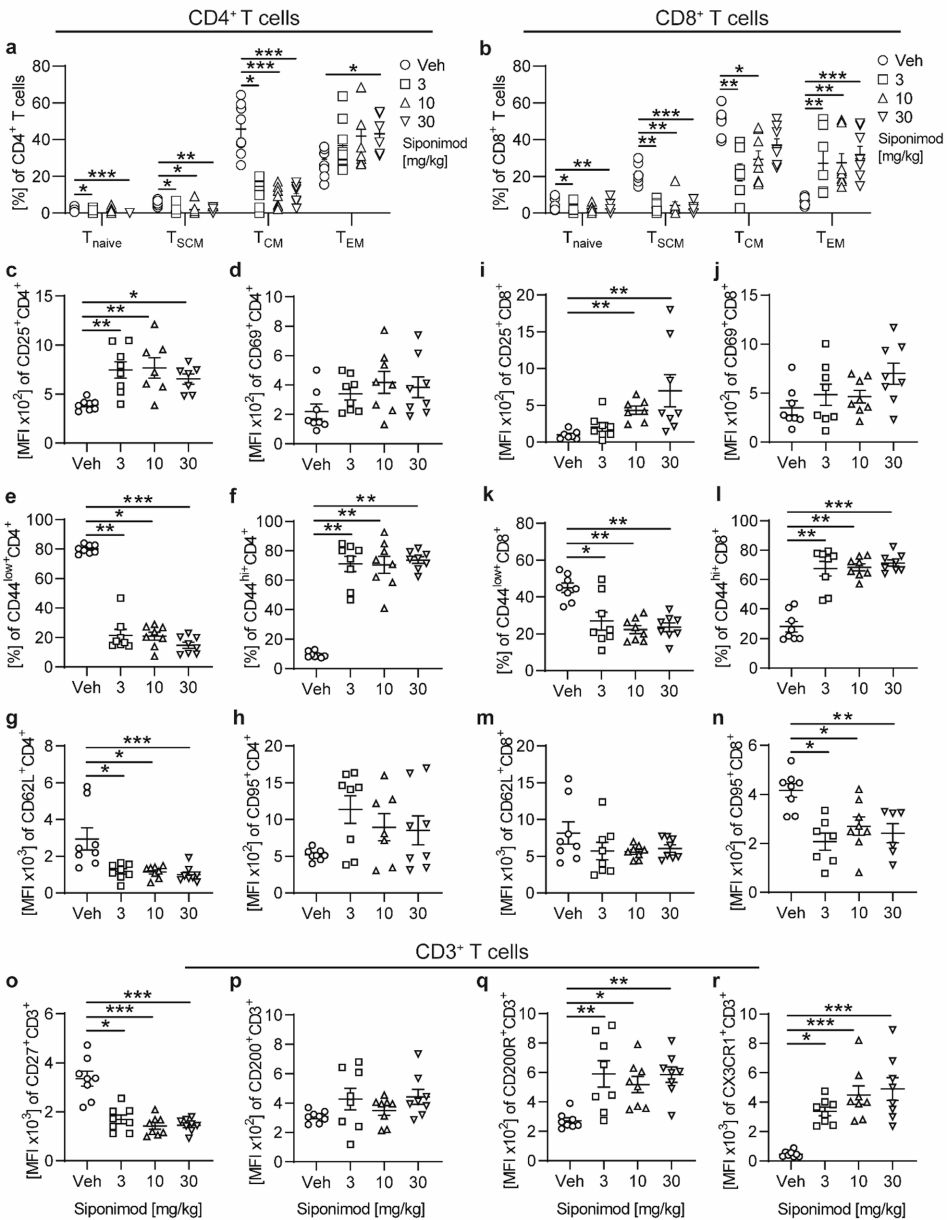
Blood T cell subsets and activation in EAE are altered upon siponimod treatment. EAE was induced by immunization with MOG peptide 35–55. Therapeutic treatment with food pellets loaded with siponimod at three different concentrations of 3, 10 or 30 mg per kg of food was initiated 20 days post immunization and maintained for at least 60 days. T cells were isolated from blood. **a, b** CD4+ and CD8+ T cell subsets (T_naive_: CD44-CD62L+CD95-; T_SCM_: CD44-CD62L+CD95+; T_CM_: CD44+CD62L+CD95+; T_EM_: CD44+CD62L-CD95+) and **c–h** CD4+ **i–n** CD8+ and **o–r** CD3+  T cell activation markers were analyzed by flow cytometry and are shown as mean fluorescence intensity (MFI); n = 8. Mean ± standard error of the mean is indicated in all graphs. Data sets are representative of three independent experiments. Asterisks indicate significant differences calculated using one-way analysis of variance Kruskal–Wallis test corrected by Dunn’s multiple comparison (*P ≤ 0.05, **P ≤ 0.01, ***P ≤ 0.001, ****P ≤ 0.0001)

Furthermore, the predominant phenotype of remaining blood T cells was shifted from naïve to more differentiated T cell subsets upon siponimod treatment (Fig. 7, Supplementary Fig. 3). As previously described, effector memory T cells (T_EM_), defined as CD44^+^CD62L^−^CD95^+^ T cells, represented the most prominent phenotype among CD4^+^ and CD8^+^ blood T lymphocytes, whereas naive T cells (T_naive_: CD44^−^CD62L^+^CD95^−^ T cells), stem cell-like memory T cells (T_SCM_: CD44^−^CD62L^+^CD95^+^ T cells) and central memory T cells (T_CM_: CD44^+^CD62L^+^CD95^+^ T cells) were largely diminished (Fig. 7a, b). Similar findings were observed in spleen (Supplementary Fig. 3a, b) and lymph nodes (not shown). Contrary to siponimod effects on the phenotype of CNS-residing T lymphocytes, siponimod treatment led to a significantly increased expression of CD25, a marker for regulatory T cells, and a decrease of CD44^low^ T lymphocytes in both blood and spleen (Fig. 7c, e, i, k, Supplementary Fig. 3c, e, i, k). CD4^+^ T cells in blood and spleen showed a significantly decreased expression of homing receptor CD62L in siponimod-treated mice compared to vehicle (Fig. 7g, Supplementary Fig. 3k). Only in T cells isolated from the spleen, the T cell activation markers CD69 and CD95 were significantly upregulated under siponimod treatment (Supplementary Fig. 3d, h, n). Notably, remaining blood T lymphocytes in siponimod-treated mice were characterized by an immunosenescent signature [16, 17] with high expression of CX3CR1 and loss of CD27 (Fig. 7o, r) while both markers remained unchanged in T lymphocytes isolated from the spleen. Furthermore, T cells in blood and spleen of siponimod-treated mice exhibited an increase of CD200R expression (Fig. 7q, Supplementary Fig. 3q), an inhibitory receptor that has been associated with T cell exhaustion [18].

### Altered blood immune cell composition under the treatment of MS with S1PR modulators

**Fig. 8:**
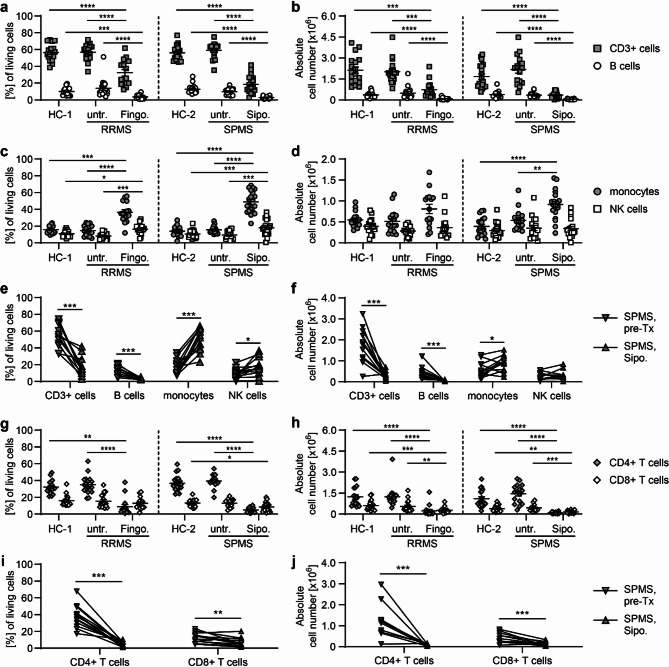
The composition of blood immune cells is differentially altered in MS patients treated with S1PR modulators. Peripheral blood mononuclear cells (PBMCs) were isolated from RRMS and SPMS patients and their age- and sex-matched healthy controls (HC-1 = healthy control cohort for RRMS, n = 18; HC-2 = healthy control cohort for SPMS, n = 20) and analyzed by flow cytometry. RRMS and SPMS patients were either untreated (untr. RRMS: n = 18; untr. SPMS: n = 16) or treated with fingolimod (Fingo: n = 15) or siponimod (Sipo: n = 20), respectively. A longitudinal analysis was additionally conduced in 13 out of 20 SPMS patients treated with siponimod. Siponimod treatment (Sipo.) was compared to parameters prior to initiation of siponimod therapy (pre-Tx). **a-j)** Relative proportion and absolute cell numbers of blood immune cell subsets. **a–d** and **g** + **h** Horizontal analysis of fingolimod and siponimod treatment compared to healthy controls. **e** + **f** and **i** + **j** Longitudinal analysis of siponimod treatment. Mean ± standard error of the mean is indicated in all graphs. Normal distribution was tested with a Shapiro–Wilk normality test. In the case of normal distribution, an ordinary one-way ANOVA test corrected with a Holm-Sidak’s multiple comparisons test was applied for horizontal comparison of different treatment groups; a two-tailed Paired t test was used for longitudinal analysis. When normal distribution was not confirmed, a one-way analysis of variance Kruskal–Wallis test corrected by Dunn’s multiple comparison was performed for horizontal analysis and a Wilcoxon matched-pairs signed rank test for longitudinal analysis. Asterisks indicate significant differences (*P ≤ 0.05, **P ≤ 0.01, ***P ≤ 0.001, ****P ≤ 0.0001)

In a translational approach, we analyzed the treatment effects of the S1PR modulators fingolimod and siponimod on frequencies and phenotypes of blood immune cells, especially T lymphocytes, in MS (Fig. 8). Fingolimod, a modulator of S1PR1, S1PR3, S1PR4 subtypes, has been approved for the therapy of RRMS, whereas the more selective S1PR1 and S1PR5 modulator siponimod is an approved treatment for active SPMS. RRMS patients under fingolimod treatment and SPMS patients under siponimod treatment were compared to independent, age- and sex-matched untreated RRMS and SPMS cohorts and healthy controls. As expected, the mean age of SPMS cohorts and their healthy control cohort HC-2 was higher than in RRMS cohorts and their respective healthy control cohort HC-1 (see Table 1 and Supplementary Table 1 for detailed demographics and clinical data). Mean treatment duration amounted to 13.60 ± 8.87 months in the siponimod treatment group and 30.7 ± 30.77 months in the fingolimod treatment group (mean ± SEM; fingolimod: n = 15, siponimod: n = 20). In addition to a horizontal analysis, siponimod treatment effects were compared longitudinally to expression patterns before treatment start in 13 out of 20 siponimod-treated SPMS patients (see Table 2 for demographics and clinical data of the longitudinal cohort). Mostly, peripheral effects of siponimod treatment on the composition of blood immune cells in MS were comparable to frequencies observed in the EAE mouse model with a prominent reduction of relative and absolute numbers of CD3^+^ cells (Fig. 8e–f). In contrast to findings in EAE, siponimod treatment of SPMS led to a significant increase of both relative frequencies and absolute cell numbers of blood monocytes. Only slight differences between treatment effects of siponimod and fingolimod were observed with a steeper decline of CD3^+^ cells and a more pronounced increase of monocytes under siponimod treatment. Equally, fingolimod and siponimod treatment induced a dramatic loss of circulating T helper cells, while absolute cell numbers of cytotoxic T cells were only slightly reduced (Fig. 8g–j). The most prominent difference between siponimod and fingolimod treatment was a significantly higher expansion of NKT cells under fingolimod treatment with NKT cells constituting almost a quarter of all CD3^+^ cells (mean ± SEM: fingolimod: 25.2 ± 4.39%, siponimod: 15.8 ± 2.29%) (Supplementary Fig. 4).

**Table 1:** Demographics and clinical characteristics of MS patient and control cohorts: horizontal analysis

Study cohorts	Number of individuals, [N (%)]	Age, [years], Mean (SD)	Disease duration, [years], Mean (SD)	EDSS, [score], Mean (SD)	Tx duration, [months], Mean (SD)
Healthy controls; cohort 1	18 (100.0)	38.5 (10.25)	n.a	n.a	n.a
Female	11 (61.1)	36.7 (10.39)	n.a	n.a	n.a
Male	7 (38.9)	41.3 (10.13)	n.a	n.a	n.a
RRMS; untreated	18 (100.0)	38.4 (10.98)	4.4 (6.48)	2.5 (1.55)	n.a
Female	11 (61.1)	36.5 (11.29)	3.2 (4.77)	2.1 (1.42)	n.a
Male	7 (38.9)	41.6 (10.52)	6.3 (8.62)	3.0 (1.71)	n.a
RRMS; fingolimod treatment	15 (100.0)	38.1 (10.24)	9.4 (7.75)	2.8 (3.10)	30.7 (30.77)
Female	10 (66.7)	37.7 (10.10)	9.3 (8.13)	2.9 (1.94)	33.5 (31.53)
Male	5 (33.3)	39.0 (11.66)	9.6 (7.83)	2.6 (2.63)	25.0 (31.89)
Healthy controls; cohort 2	20 (100.0)	54.2 (11.41)	n.a	n.a	n.a
Female	11 (55.0)	51.8 (9.13)	n.a	n.a	n.a
Male	9 (45.0)	57.0 (13.72)	n.a	n.a	n.a
SPMS; untreated^a^	16 (100.0)	56.9 (9.95)	18.4 (10.67)	4.8 (1.58)	n.a
Female	11 (68.8)	54.1 (7.64)	17.6 (8.13)	4.5 (1.53)	n.a
Male	5 (31.3)	63.0 (12.55)	19.8 (15.68)	5.6 (1.56)	n.a
SPMS; siponimod treatment	20 (100.0)	55.5 (10.32)	15.3 (12.36)	5.0 (1.54)	13.6 (8.87)
Female	11 (55.0)	55.3 (9.93)	18.0 (15.51)	4.7 (1.33)	14.0 (7.50)
Male	9 (45.0)	55.8 (11.39)	11.9 (6.22)	5.3 (1.79)	13.1 (10.78)

EDSS, Expanded Disability Status Scale; MS, multiple sclerosis; n.a., not applicable; RRMS, relapsing remitting MS; Tx duration, fingolimod or siponimod treatment duration; SPMS, secondary progressive MS; SD, standard deviation

^a^Data on disease duration was not available in 1 of 16 untreated SPMS patients

**Table 2:** Demographics and clinical characteristics of the longitudinal siponimod treatment cohort

Longitudinal siponimod treatment cohort	Number of SPMS patients, N (%)	Age (pre-Tx), [years], Mean (SD)	Disease duration (pre-Tx), [years], Mean (SD)	Sipo-Tx duration, [months], Mean (SD)	EDSS (pre-Tx), [score], Mean (SD)	EDSS (Sipo-Tx), [score], Mean (SD)
All patients	13 (100.1)	55.2 (10.57)	14.4 (12.99)	12 (8.7)	4.9 (1.36)	5.0 (1.40)
Female	8 (61.5)	55.0 (8.55)	15.8 (16.25)	12 (6.2)	4.5 (1.25)	4.6 (1.18)
Male	5 (38.5)	55.6 (14.38)	12.1 (5.79)	12 (12.7)	5.5 (1.41)	5.7 (1.57)

EDSS, Expanded Disability Status Scale; MS, multiple sclerosis; pre-Tx, before siponimod treatment start; RRMS, relapsing remitting MS; Sipo-Tx, siponimod treatment; SPMS, secondary progressive MS; SD, standard deviation

### Predominance of regulatory and immunosenescent blood T cell phenotypes in MS treated with S1PR modulators

**Fig. 9:**
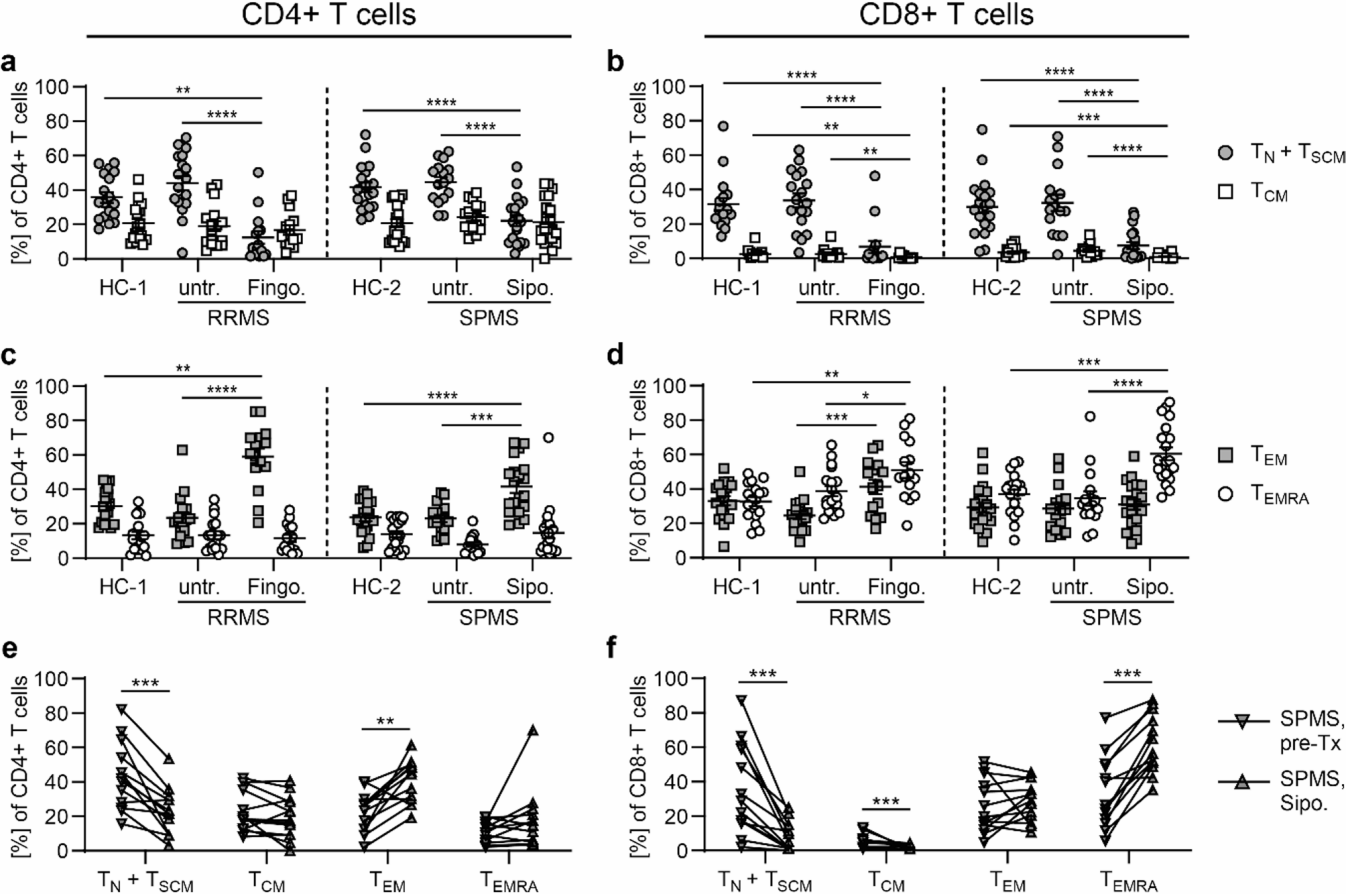
CD4+ effector memory T cells (T_EM_) and CD8+ terminally differentiated effector memory T cells (T_EMRA_) represent predominant T cell phenotypes under siponimod treatment. Peripheral blood mononuclear cells (PBMCs) were isolated from RRMS and SPMS patients and their age- and sex-matched healthy controls (HC-1 = healthy control cohort for RRMS, n = 18; HC-2 = healthy control cohort for SPMS, n = 20) and analyzed by flow cytometry. RRMS and SPMS patients were either untreated (untr. RRMS: n = 18; untr. SPMS: n = 16) or treated with fingolimod (Fingo: n = 15) or siponimod (Sipo: n = 20), respectively. A longitudinal analysis was additionally conducted in 13 out of 20 SPMS patients treated with siponimod. Siponimod treatment (Sipo.) was compared to parameters prior to initiation of siponimod therapy (pre-Tx). **a–f** Frequency of T cell subsets representing different stages of T cell differentiation: T_N_ + T_SCM_: CD45RO-CCR7+; T_CM_: CD45RO+CCR7+; T_EM_: CD45RO+CCR7-; T_EMRA_: CD45RO-CCR7-; **a, c, e** T helper cells (CD4+CD8-CD3+ cells); **b, d, f** cytotoxic T cells (CD4-CD8+ CD3+ cells); **a–d** Horizontal analysis of fingolimod and siponimod treatment compared to controls; **e, f** Longitudinal analysis of siponimod treatment. Mean ± standard error of the mean is indicated in all graphs. Normal distribution was tested with a Shapiro–Wilk normality test. In the case of normal distribution, an ordinary one-way ANOVA test corrected with a Holm-Sidak’s multiple comparisons test was applied for horizontal comparison of different treatment groups; a two-tailed Paired t test was used for longitudinal analysis. When normal distribution was not confirmed, a one-way analysis of variance Kruskal–Wallis test corrected by Dunn’s multiple comparison was performed for horizontal analysis and a Wilcoxon matched-pairs signed rank test for longitudinal analysis. Asterisks indicate significant differences (*P ≤ 0.05, **P ≤ 0.01, ***P ≤ 0.001, ****P ≤ 0.0001)

**Fig. 10:**
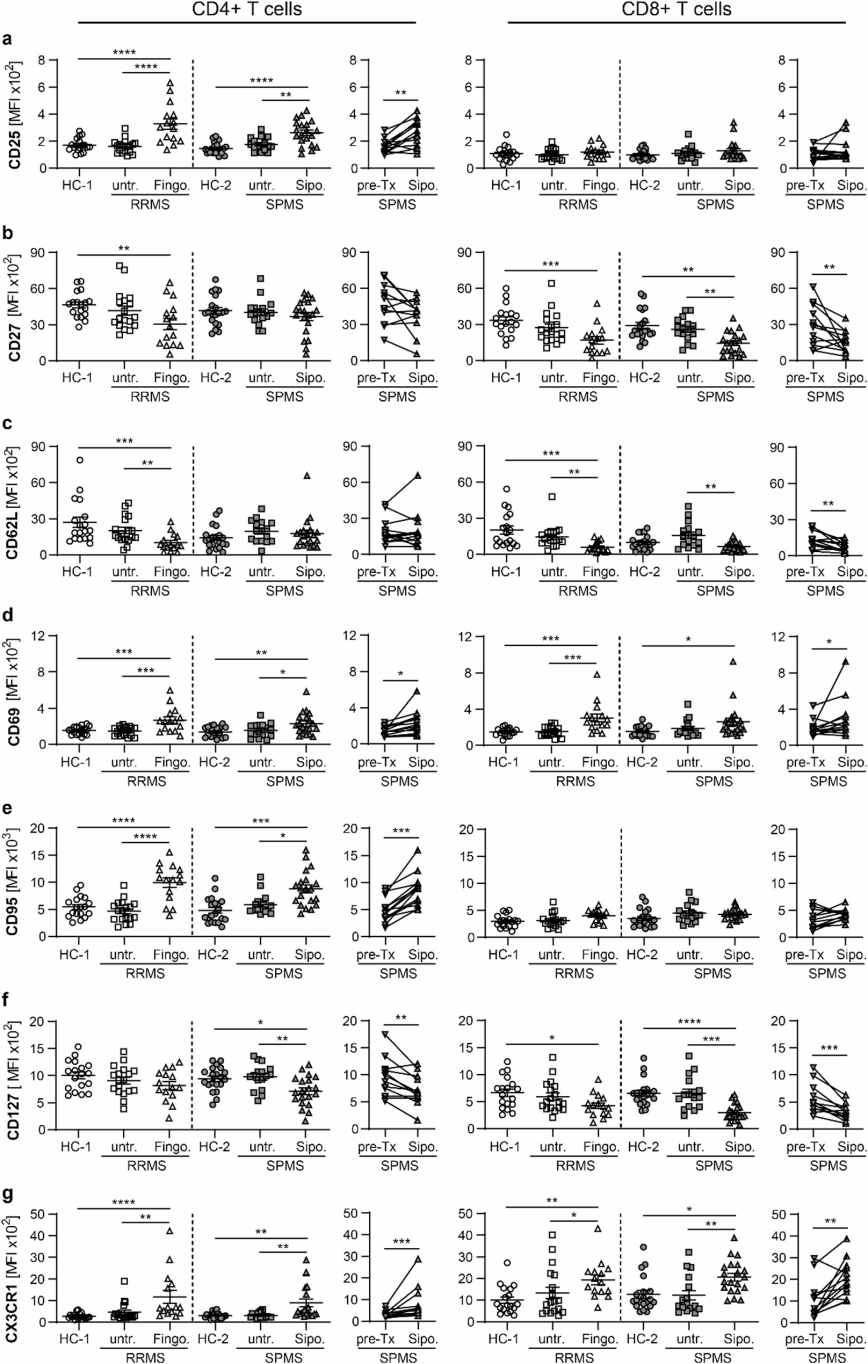
Blood T lymphocytes exhibit an immunosenescent signature in MS patients treated with S1PR modulators. Peripheral blood mononuclear cells (PBMCs) were isolated from RRMS and SPMS patients and their age- and sex-matched healthy controls (HC-1 = healthy control cohort for RRMS, n = 18; HC-2 = healthy control cohort for SPMS, n = 20) and analyzed by flow cytometry. RRMS and SPMS patients were either untreated (untr. RRMS: n = 18; untr. SPMS: n = 16) or treated with fingolimod (Fingo; n = 15) or siponimod (Sipo; n = 20), respectively. A longitudinal analysis was additionally conduced in 12–13 out of 20 SPMS patients treated with siponimod. Siponimod treatment (Sipo.) was compared to parameters prior to initiation of siponimod therapy (pre-Tx). Expression of cell surface markers of T helper (CD4+ T cells) cells and cytotoxic T cells (CD8+ T cells) are shown as mean fluorescence intensity (MFI). Mean ± standard error of the mean is indicated in all graphs. Normal distribution was tested with a Shapiro–Wilk normality test. In the case of normal distribution, an ordinary one-way ANOVA test corrected with a Holm-Sidak’s multiple comparisons test was applied for horizontal comparison of different treatment groups; a two-tailed Paired t test was used for longitudinal analysis. When normal distribution was not confirmed, a one-way analysis of variance Kruskal–Wallis test corrected by Dunn’s multiple comparison was performed for horizontal analysis and a Wilcoxon matched-pairs signed rank test for longitudinal analysis. Asterisks indicate significant differences (*P ≤ 0.05, **P ≤ 0.01, ***P ≤ 0.001, ****P ≤ 0.0001)

Similar to our findings in murine EAE, taking species differences in T cell differentiation to account, we observed a dramatic shift from naive to more differentiated, immunosenescent T cell subsets in MS patients treated with fingolimod or siponimod (Fig. 9). T cell subsets representing different stages of T cell differentiation were defined by their CD45RO and CCR7 expression (T_N_ + T_SCM_: CD45RO^−^CCR7^+^; T_CM_: CD45RO^+^CCR7^+^; T_EM_: CD45RO^+^CCR7^−^; T_EMRA_: CD45RO^−^CCR7^-^). As previously reported, T_EM_ cells represented the most frequent blood T cell subset in RRMS patients treated with fingolimod, whereas the more differentiated, less proliferatively active T_EMRA_ subset was most abundant in SPMS patients treated with siponimod, indicating an even more pronounced shift to immunosenescence under siponimod treatment (Data not shown). In a further characterization of T cells (Fig. 10), we observed a significant loss of CD27 expression and a steep upregulation of CX3CR1 expression, especially in cytotoxic T cells, as indications for an immunosenescent T cell phenotype (Fig. 10b, g). Furthermore, an upregulation of activation markers CD69 and CD95 and a downregulation of homing receptor CD62L was shown under siponimod and fingolimod treatment (Fig. 10c–e). In T helper cells, an increased expression of CD25 and a downregulation of CD127 were indicative of an increased proportion of regulatory blood T cells (Fig. 10a, f). In summary, our findings demonstrate that siponimod treatment in SPMS leads to a marked reduction in circulating blood T cell frequencies, with a predominance of immunosenescent T cell subsets and an increase of regulatory T cell functions. Overall, the immunomodulatory effects of siponimod on peripheral blood immune cells in SPMS patients closely mirrored those observed in our chronic EAE mouse model.

### Blood biomarkers for neuroaxonal damage and progression

For further evaluation whether neuroprotective effects we observed in therapeutic treatment of chronic EAE could be translated from the mouse model to siponimod treatment effects in SPMS, we analyzed the expression of serum NfL (sNfL), a biomarker for neuroaxonal damage, and of serum GFAP (sGFAP) as surrogate marker for progression in our MS patient cohorts and their age-matched healthy controls with a SIMOA multiplex assay (Supplementary Fig. 5). An analysis of sNfL and sGFAP levels in healthy controls showed an age dependency that was even more pronounced in a cohort of untreated RRMS and SPMS patients, especially after the age of forty (Supplementary Fig. 5 a–f). Only in SPMS patients treated with siponimod, a reduction of median sGFAP concentrations to values seen in the healthy control cohort could be observed, while median sNFL levels were not different to untreated MS patients (median sGFAP levels: HC-1: 94.9 pg/ml; untr. RRMS: 108.4 pg/ml; fingolimod-treated RR-MS: 100.8 pg/ml; HC-2: 104.0 pg/ml; untr. SPMS: 167.9 pg/ml; siponimod-treated SPMS: 101.9 pg/ml). Longitudinal analysis of siponimod treatment also showed a decrease of sGFAP levels under siponimod treatment (median sGFAP levels: pre-Tx: 175.1 pg/ml; under siponimod treatment: 108.7 pg/ml). Additionally, we determined soluble TREM2 (sTREM2) serum levels as potential marker for microglial activation in the context of progression with a SIMOA assay. However, no significant differences in sTREM2 serum levels were observed between treatment groups (Supplementary Fig. 5g, h).

## Discussion

In this manuscript, we are reporting on the effect of siponimod in progression of chronic CNS inflammation. Using an animal model of chronic EAE, we could show that therapeutic siponimod treatment promotes a clinically significant resolution of already established inflammatory demyelinating lesions with a decrease of lesional demyelinated white matter areas and reduced plasma NfL levels as surrogate parameter for neuroaxonal damage. This is in accordance with findings from previous animal studies and strongly supports an earlier MRI study showing improved lesion resolution in active SPMS patients treated with siponimod [19]. However, we initiated siponimod treatment in the early chronic disease phase. It remains unclear whether a similar disease amelioration could be observed, if siponimod treatment was started at a later time point. We also observed an increased number of Olig2-positive cells in spinal cord white matter section under siponimod treatment. Recent studies investigated the effect of siponimod on remyelination in various demyelinating models, showing a more protective effect on differentiating oligodendrocytes rather than inducing OPC proliferation [20, 21]. Of importance, siponimod also binds to S1PR5, which in the CNS is almost exclusively expressed in oligodendrocytes. Endothelial cells in the CNS also express S1PR5. Therefore, it remains unclear to what extent the observed effects were mediated by oligodendrocytes. Nevertheless, markers associated with regenerative phenotypes of microglia were also detected in these studies [21, 22], indicating that this effect might be mediated by a modulation of phagocyting microglia, an aspect which needs further investigations.

Moreover, the beneficial clinical effect of siponimod treatment was associated with a reduced number, but even more so reduced activation and proinflammatory differentiation of CNS-infiltrating T cells. Furthermore, siponimod treatment reduced the proinflammatory response of microglia, most prominently observed by a robust reduction of microglial MHC class II expression in vivo. In this regard, a recent neuropathological study of microglia subtypes in autopsy tissue from MS patients, MHC class II-expressing, non-ramified microglia were typically observed at margins of chronic MS lesions, whereas this microglia phenotype was largely absent from acute MS lesions [23], thus supporting the hypothesis that highly MHC class II-expressing microglia are functionally linked to neurodegenerative chronic progression of MS. Taken together, our in vivo findings thus support that siponimod treatment is capable of controlling established ongoing CNS inflammation, potentially through a direct or indirect microglia-mediated mechanism of action.

In an approach to dissect the respective effect of siponimod on CNS-infiltrating T cells versus microglia, we identified that in vitro siponimod-preconditioned microglia are less prone to activation by IFNγ, the hallmark cytokine produced by encephalitogenic T cells [24]. Several studies have previously reported a direct in vitro effect of siponimod on proinflammatory responses of microglia [25, 26]. In particular, activation of microglia with LPS and TNFα/IL-17 was inhibited by siponimod. In contrast to these observations, we did not detect any changes in LPS or TNFα-activated microglia upon siponimod pretreatment.

Of note, IFNγ signaling induces a typical signature of IFNγ-regulated genes, among them MHC class II. In brain tissue of MS patients, an upregulation of IFNγ-induced genes has been described in microglia located at the border of chronic white matter lesions. Notably, these IFNγ-induced genes were involved in detrimental, proinflammatory as well as protective, anti-oxidative pathways [27]. In contrast to the significant downregulation of MHC class II expression in microglia shown in chronic EAE under siponimod treatment, we did not observe an inhibition of INFγ-induced MHC class II upregulation in primary microglial cultures through siponimod pretreatment. However, when siponimod-treated myelin-specific T cells were cultured with IFNγ-preconditioned primary microglia, we observed a reduction of MHC class II expression in microglia. In return, when microglia preconditioned with siponimod were used as antigen presenting cells for the activation of myelin-specific T cells, we observed a strong reduction in proliferation, activation and proinflammatory differentiation of the responding T cells. These findings support that siponimod’s mode of action involves the regulation of reciprocal interactions between T cells and microglia. In conjunction, these findings support the concept that siponimod substantially limits the proinflammatory, self-perpetuating interaction of microglia and CNS-infiltrating T cells, as one assumed key correlate of MS progression [28].

Having established these reciprocal inhibitory effects on microglia and T cells within the CNS, we next focused on the effect of siponimod on peripheral immune cells, particularly T cells prior to their infiltration. In mice with established EAE, we observed a lower abundance of T and B cells in the blood, closely resembling the situation in sipominod-treated MS patients. In contrast, in secondary lymphoid organs, we only found a lower number of T cells while B cell frequencies remained unchanged or even increased. This differential effect of siponimod on T and B lymphocytes might be indicative of an impaired T cell proliferation under siponimod treatment in addition to a retention in lymph nodes due to an inhibitory effect on lymphocyte migration. Furthermore, these experimental findings may have fundamental implications for the recovery of B versus T cells upon succession of treatment with a potentially faster recovery of B cells. Besides these antimigratory effects on lymphocytes, siponimod also altered the composition of T cells in regard to maturation and differentiation and induced a shift towards an immunosenescent, regulatory phenotype. Taken together these findings in the blood and secondary lymphoid organs in experimental MS closely reflect the human situation and largely explain how these peripheral effects connect to the observed, lowered abundance and activation of T cells infiltrating the CNS.

Lastly, we wanted to connect our experimental findings as closely as possible to the situation in siponimod treated individuals. Investigating age- and sex-matched cohorts of patients with MS, we could confirm that siponimod treatment led to a strong decline of T and B lymphocytes in the peripheral blood of siponimod-treated SPMS patients and also confirmed the inhibitory effects on T cell differentiation resulting in a shift of T cell subsets towards immunosenescent, exhausted T cell phenotypes. To further evaluate whether the evidence for neuroprotective effects obtained in mice and in in vitro models could be translated to the siponimod treatment effects in MS, we included an analysis of serum surrogate markers for progression and neuroaxonal damage. Although the small sample size of our MS patients and control cohorts represents a limitation for the interpretation of the biomarker expression, we observed a reduction of median serum GFAP levels under siponimod treatment, hinting at a potential beneficial effect on astrocyte-mediated pathomechanisms of progression besides the reduced CNS immune cell infiltration.

Taken together, these experimental and human findings establish and confirm therapeutically desirable properties of siponimod on various aspects of established CNS chronic inflammation. Specifically, siponimod treatment leads to an inhibition of detrimental T cell-microglia interactions associated with neurodegenerative processes through a dual central and peripheral mechanism of action. On the one hand, siponimod protects microglia from being activated, particularly via T cell-produced IFNγ, and on the other hand strongly reduces the likelihood that peripheral immune cells differentiate in a proinflammatory manner as a prerequisite of their microglia-activating properties. Based on these complementary properties, siponimod appears particularly suitable to control CNS-compartmentalized inflammatory circuits between CNS-resident cells and CNS-infiltrating immune cells and thus rightfully serves as the current gold standard in treatment of SPMS.

## Methods

### Mice

Wild-type C57BL/6J mice were purchased from Charles River (Sulzfeld, Germany). MOG p35-55 TCR transgenic 2D2 mice were kindly provided by Dr. Kuchroo (Boston, USA).

### Sex as a biological variable

For all active EAE experiments, female mice were examined. For studies using human blood samples, MS patients under treatment with S1P receptor inhibitors were sex-matched to their untreated and healthy controls.

### Siponimod treatment of mice

Siponimod treatment was orally administered via vehicle food pellets loaded with siponimod at three different concentrations of 3, 10 or 30 mg per kg of food and provided to mice at libitum. The diet of all mice was changed to vehicle food a week before immunization. 20 days post immunization (dpi) mice were assigned to treatment groups for an equal distribution of clinical EAE severity across all treatment groups. Siponimod treatment was initiated and maintained for at least 60 days until mice were sacrificed for analysis between 80 to 92 dpi. In total, three independent experiments using the chronic EAE model were performed (experiment 1: 13 mice/treatment group; experiment 2: 10 mice/treatment group; experiment 3: 13 mice/treatment group) resulting in a pooled group size of 36 mice/treatment. In experiment 2 as well as in experiment 3, one mouse belonging to the 10 mg/kg siponimod treatment group died from causes unrelated to the disease model, resulting in a pooled group size of 34 mice for this treatment group. Clinical effects of siponimod treatment were consistent across all three individual experiments.

### Measurement of siponimod plasma levels in EAE mice

Siponimod plasma levels of mice were determined using Liquid Chromatography coupled to tandem Mass Spectrometry (LC–MS/MS).

### EAE induction and scoring

Female wild-type C57BL/6J mice were immunized subcutaneously with 75 µg MOG peptide (p)35–55 MEVGWYRSPFSRVVHLYRNGK (Auspep) emulsified in complete Freund’s adjuvant (Sigma-Aldrich) containing 250 µg inactivated *Mycobacterium tuberculosis* H37 Ra (BD Bioscience) followed by intraperitoneal injections of 200 ng of *Bordetella pertussis* toxin (Sigma-Aldrich) on the day of immunization and two days thereafter. EAE severity was assessed daily and scored on a scale from 0 to 5 as follows: 0 = no clinical signs; 1.0 = tail paralysis; 2.0 = hindlimb paresis; 3.0 = severe hindlimb paresis; 4.0 = paralysis of both hindlimbs; 4.5 = hindlimb paralysis and beginning forelimb paresis; and 5.0 = moribund/death.

### Histology and immunohistochemistry

Mice were transcardially perfused with PBS followed by 4% paraformaldehyde (PFA) and tissue was paraffin embedded. One-micrometre thick slices were stained with haematoxylin and eosin and Luxol fast blue/periodic acid-Schiff. Oligodendroglial lineage cells, T cells, B cells, macrophages and microglia were detected by immunohistochemistry with an avidin–biotin technique using antibodies specific for CD3 (SP7; DCS Innovative Diagnostik-Systeme), CD45R/B220 (RA3-6B2; BD Biosciences), Olig2 (polyclonal; IBL—America), Mac-3 (M3/84; BD Biosciences) and Iba1 (polyclonal; Fujifilm), respectively. For more information on antibodies for immunohistochemistry, see Supplementary Table 2. Histological sections were captured using a digital camera (DP71; Olympus Europa) mounted on a light microscope (BX51; Olympus Europa). The percentage of demyelinated white matter was calculated in relation to the total area of white matter after Luxol Fast Blue/Periodic Acid Schiff staining using cellSens Dimension software (Olympus Europa). Overall spinal cord inflammation was evaluated by haematoxylin and eosin staining and assessed on a scale from 0 to 3 as follows: 0 = no infiltration; 1 = minor infiltration; 2 = moderate infiltration; 3 = pronounced infiltration. Inflammatory cells were quantified in the white matter after appropriate immunohistochemical staining at × 400 magnification using an ocular counting grid and are shown as cells/mm^2^. At least eight spinal cord cross sections per animal were taken for each analysis.

### Human samples

Blood donors for the analysis of peripheral blood mononuclear cells and serum were recruited from MS patients treated at the Department of Neurology, University Medical Center Göttingen, and from healthy volunteers.

### Isolation of human and murine leukocytes and CNS-resident cells

Peripheral blood mononuclear cells (PBMC) from human study subjects were isolated via Ficoll gradient centrifugation. For the isolation of murine leukocytes and plasma, blood samples were drawn from the facial vein of mice and collected in EDTA-containing tubes. Single cell suspensions of murine lymphoid tissues were generated by gentle dissection and passing through 70 µm cell strainer (Greiner Bio-One). Brain and spinal cord tissues were isolated from mice upon perfusion with phosphate-buffered saline (PBS) and were dissociated to single cells using the Multi Tissue Dissociation Kit (Miltenyi Biotec). Infiltrating myeloid cells were distinguished from CNS-resident microglia by flow cytometry (Supplementary Fig. 6).

### Generation of primary microglia

To generate primary microglia, brain cells of new-born to two-day-old C57BL/6J mice were isolated enzymatically using 2.5% trypsin (Pan Biotech) and 0.4 mg DNAse I (Roche). First, a mixed glial cell culture was generated by seeding the cells in DMEM containing 10% foetal calf serum, 1% GlutaMax™, 100 U/ml penicillin, and 100 µg/ml streptomycin and cultivating them at 37° C and 5% CO_2_ until confluency was reached. To obtain enriched microglia cultures, cells were thereafter stimulated for five days with medium containing 30% conditioned L929 cell supernatant (DMEM, 30% L929 supernatant, 10% foetal calf serum, 100 U/ml penicillin, and 100 µg/ml streptomycin). Primary microglia were harvested by gentle shaking at 90 rpm for 30 min at 37 °C to separate microglial cells from other glia cell. Cultures contained > 95% microglial cells verified by flow cytometry.

### Co-culture of microglia and T cells in vitro

Microglia (0.3 × 10^5^ cells/well) were plated into 96-well flat-bottom plates and stimulated with 1µM siponimod or DMSO control, respectively for 18h. Where indicated 10 ng/ml IFNy was added additionally. After 18h, microglia were washed twice and 0.5 × 10^5^ MACS-purified (Pan T cell Isolation Kit, Miltenyi, Bergisch Gladbach, Germany) carboxyfluorescein succinimidyl ester (CFSE)-stained (CFSE Cell Division Tracker Kit, BioLegend) or unstained T cells from 2D2 mice were added per well. 72 h after co-culture in the presence of MOG p35-55, T cell proliferation and differentiation were evaluated by flow cytometry and/or ELISA.

### Enzyme-linked immunosorbent assays (ELISA)

Production of murine IL-17 was measured using ELISA MAX Standard Set kits (BioLegend). Murine IL-2 was measured using DuoSet ELISA kits (R&D Systems). Absorbance was determined at 450 nm with subtraction of a 540 nm reference wavelength on iMark™ microplate reader (Bio-Rad laboratories Inc.).

### Flow cytometry of human and murine samples

In all flow cytometry experiments, pregating included definition of cells by size and exclusion of doublets. Dead cells were stained with the Zombie Fixable Viability™ Kit (BioLegend).

Human PBMC were stained for lineage markers using the following antibodies: CD3 (UCHT1; BD Biosciences), CD4 (RPA-T4; BioLegend), CD8 (HIT8a; BioLegend), CD14 (M5E2; BioLegend), CD19 (HIB19; BioLegend) and CD56 (B159; BD Bioscience). T cell activation and differentiation was analyzed using: CD25 (BC-96; BioLegend), CD27 (M-T271, BioLegend), CD95 (DX2; BioLegend), CD45 RA (HI100; BD Biosciences), CD45RO (UCHL1; BioLegend), CD62L (DREG-56; BioLegend), CD69 (FN50; BioLegend), CD127 (HIL-7R-M21; BD Biosciences), CD197 (CCR7; 150,503; BD Biosciences) and CX3CR1 (K0124E1; BioLegend).

Composition of murine immune cells was analysed using the following antibodies: CD3 (145-2C11; BD Bioscience), CD4 (RM4-5; BioLegend), CD8 (53–6.7; BioLegend), CD19 (6D5; BioLegend), CD20 (SA275A11; BioLegend), CD11b (M1/70; BioLegend), CD11c (N418; BioLegend), CD45 (30-F11; BioLegend), Ly6C (HK1.4; BioLegend) and Ly6G (1A8: BioLegend). Microglia activation, differentiation and molecules involved in antigen presentation were determined using: CD68 (FA-11; BioLegend), CD69 (H1.2F3; BioLegend), CD80 (16-10A1; BD Bioscience), CD86 (GL-1; BioLegend), MHCII (AF6-120.1; BioLegend) and PD-L1 (MIH5; eBioscience). T cell activation and differentiation was assessed using: CD25 (PC61; BioLegend), CD27 (LG.3A10; BioLegend), CD44 (IM7; BioLegend), CD62L (MEL-14; BioLegend), CD69 (H1.2F3; BioLegend), CD95 (Jo2; BD Biosciences), CD200 (OX-90; BioLegend), CD200R (OX110; BioLegend), CX3CR1 (SA011F11; BioLegend). Fc receptors were blocked using monoclonal antibody specific for murine or human CD16/CD32 (Murine TruStain FcX; Human TruStain FcX; BioLegend), respectively. Intracellular proteins were analysed using the BD PhosFlow protocol and analysed using the following antibodies: STAT1 (pY701) (4a; BD Bioscience). For more information on antibodies for flow cytometry, see Supplementary Tables 3 and 4. Samples were acquired on a BD LSR Fortessa (BD Bioscience). All data evaluation was performed using FlowJo software (FlowJo LLC, Ashland, USA).

### NFL, GFAP and sTREM2 measurements

Mouse EDTA plasma samples and human serum samples were stored at -80°C until analysis. Biomarker measurements were performed using Single Molecule Arrays (SIMOA) on a SIMOA HD-1 analyzer with commercial kits according to the manufacturer’s protocols (Quanterix, MA USA). Mouse plasma NfL levels were measured in unicates with the bead-based NF-Light advantage kit. In human serum samples, NfL and GFAP measurements were performed with the multiplex bead-based SIMOA Neurology 2-Plex B advantage kit and sTREM2 levels were determined using the SIMOA sTREM2 advantage kit. For analysis of NfL, GFAP and sTREM2 concentrations in human serum samples, mean values of technical duplicates were determined.

### Quantitative PCR

RNA was isolated using the RNeasy mini kit (Qiagen) and transcribed into cDNA using the QuantiNova Reverse Transcription kit (Qiagen). Quantitative (q) PCR was performed using 500 nM Primer and qPCRBIO SyGreen in a total volume of 10 µl on a QuantStudio 7. Primers specific for S1PR1 (forward: 5'-TCA TAG TCC GGC ATT ACA ACT A-3'; reverse: 5'-GTG TGA GCT TGT AAG TGG TG-3'), S1PR2 (forward: 5'-ATG GGC GGC TTA TAC TCA GAG -3'; reverse: 5'-GCG CAG CAC AAG ATG ATG AT-3'), S1PR3: (forward: 5'-TTG TGG TGA GTG TGT TCA TTG -3', reverse: 5'-TCA CTT GCA GAG GAC CCC GTT C -3'), S1PR4 (forward: 5'-CTC CAA GGG CTA TGT GCT CT-3', reverse: 5'-ATT GGC TCG GAC CAC TCT AA-3'), S1PR4 (forward: 5'-GCC TGG TGC CTA CTG CTA CAG-3', reverse: 5'-CCT CCG T?G CTG GGT ATT TCC-3') and housekeeping gene B2M (forward 5’- CGGCCTGTATGCTATCCAGA-3’, reverse: 5’ GGGTGAATTCAGTGTGAGCC-3’) were purchased from Eurofins Genomics. qPCR was performed at 95°C desaturating and 68°C annealing temperature for 30 s and 40 cycles with subsequent melt-curve analysis. Primer specificity was validated by product size using a 2% Agarose gel containing GelRed and UV-light illumination. Samples were analysed in triplicate and considered valid when cycle threshold (Ct) < 35 and standard deviation of Ct < 0.5. The relative expression was determined in comparison to the control-treated group.

### Statistical analysis

Statistics were calculated using GraphPad Prism 6 or GraphPad Prism 10.5.0. For the analysis of ex vivo experiments, Gauss distribution was tested via Shapiro–Wilk normality test if n > 6; for experiments with n ≤ 6, a non-Gauss distribution was assumed. For the analysis of in vitro experiments, Gauss distribution was assumed. The respective statistical comparisons used are indicated in the figure legendsFor the analysis of human PBMC and serum biomarker expression, Gauss distribution was tested in all samples using a Shapiro–Wilk normality test with a significance level alpha = 0.05. In the case of normal distribution, an ordinary one-way ANOVA test corrected with a Holm-Sidak’s multiple comparisons test was applied for horizontal comparison of different treatment groups; a two-tailed Paired t test was used for longitudinal analysis (comparison of parameters before and after siponimod treatment). When normal distribution was not confirmed, a one-way analysis of variance Kruskal–Wallis test corrected by Dunn’s multiple comparison was performed for horizontal analysis and a Wilcoxon matched-pairs signed rank test was used for longitudinal analysis.

## Supplementary Information

Below is the link to the electronic supplementary material.

Supplementary Material 1.

## Data Availability

Data is provided within the manuscript or supplementary information files.
