# Supplementary material for: Siponimod inhibits disease-associated microglia-T cell interactions in chronic experimental autoimmune encephalomyelitis
Source: Acta Neuropathol Commun. 2025 Dec 1;13:247. doi: 10.1186/s40478-025-02136-3 (PMC12670806; doi:10.1186/s40478-025-02136-3)
Supplement: Supplementary file 1 — Supplementary Material 1. [file 40478_2025_2136_MOESM1_ESM.docx]

**SUPPLEMENTARY**

**
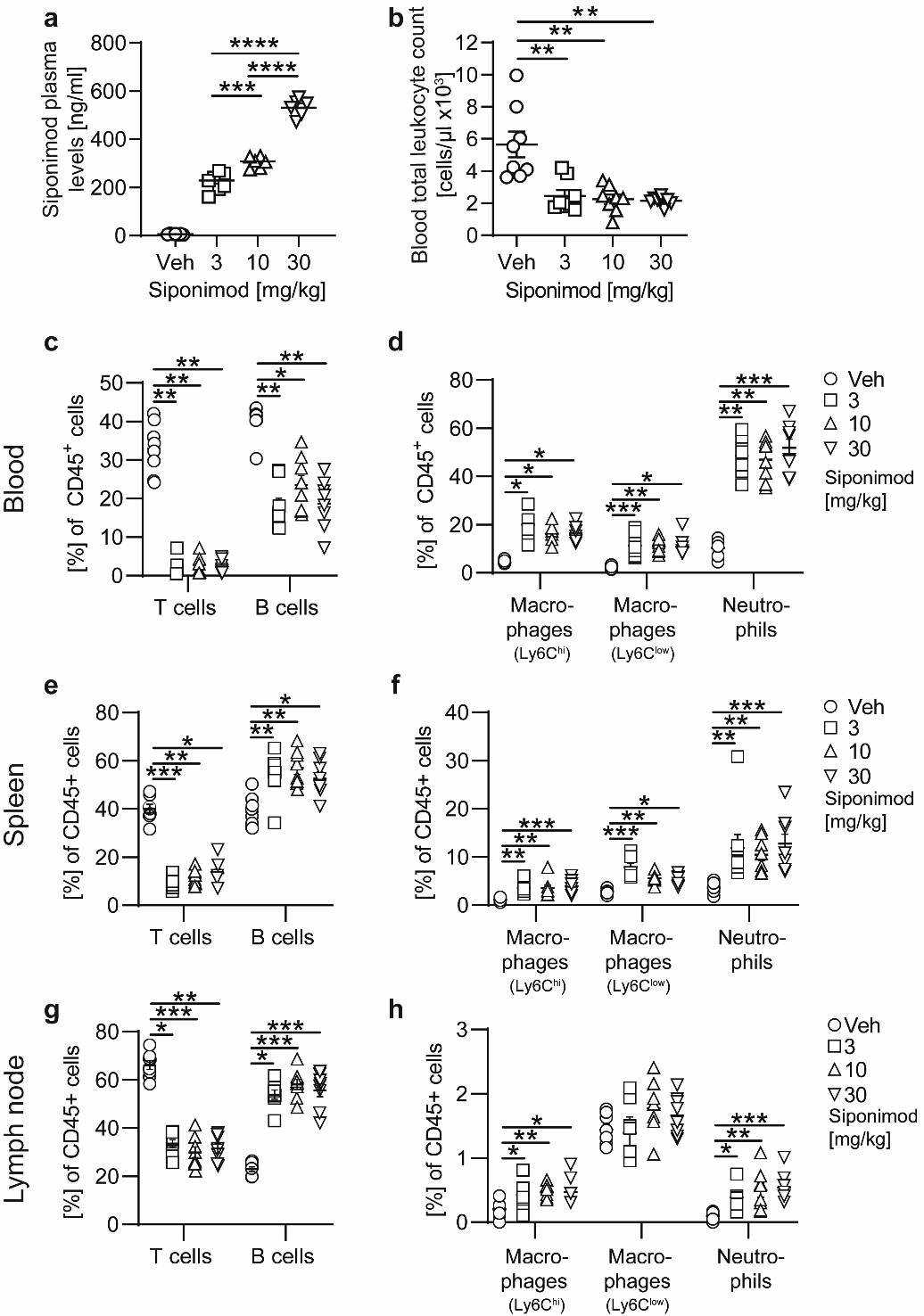
**

**Supplementary Figure 1. Siponimod treatment differentially alters the frequency of immune cells in blood versus lymphoid organs.** EAE was induced by immunization with MOG peptide 35-55. Therapeutic treatment with food pellets loaded with siponimod at three different concentrations of 3, 10 or 30 mg per kg of food was initiated 20 days post immunization for 60 days. **a)** Siponimod plasma levels, **b)** total leukocyte cell count in blood. Immune cells were isolated from **c-d)** blood, **e-f)** spleen**, g-h)** lymph nodes and composition of immune cells (T cells: CD45+CD11b-CD3+; B cells: CD45+CD11b-CD19+; macrophages: CD11b+CD45^hi^Ly6C^low^; macrophages: CD11b+CD45^hi^Ly6C^hi^; neutrophils: CD11b+CD45^hi^Ly6C+Ly6G+) were analyzed by flow cytometry and are shown as frequencies); n = 8. Mean ± standard error of the mean is indicated in all graphs. **a)** n = 7; **b -h)** Data sets are representative of three independent experiments. Asterisks indicate significant differences calculated using **a-b)** one-way analysis of variance corrected by Holm-Sidak or **c-h)** one-way analysis of variance Kruskal-Wallis test corrected by Dunn’s multiple comparison (*P ≤ 0.05, **P ≤ 0.01, ***P ≤ 0.001, ****P ≤ 0.0001).

**
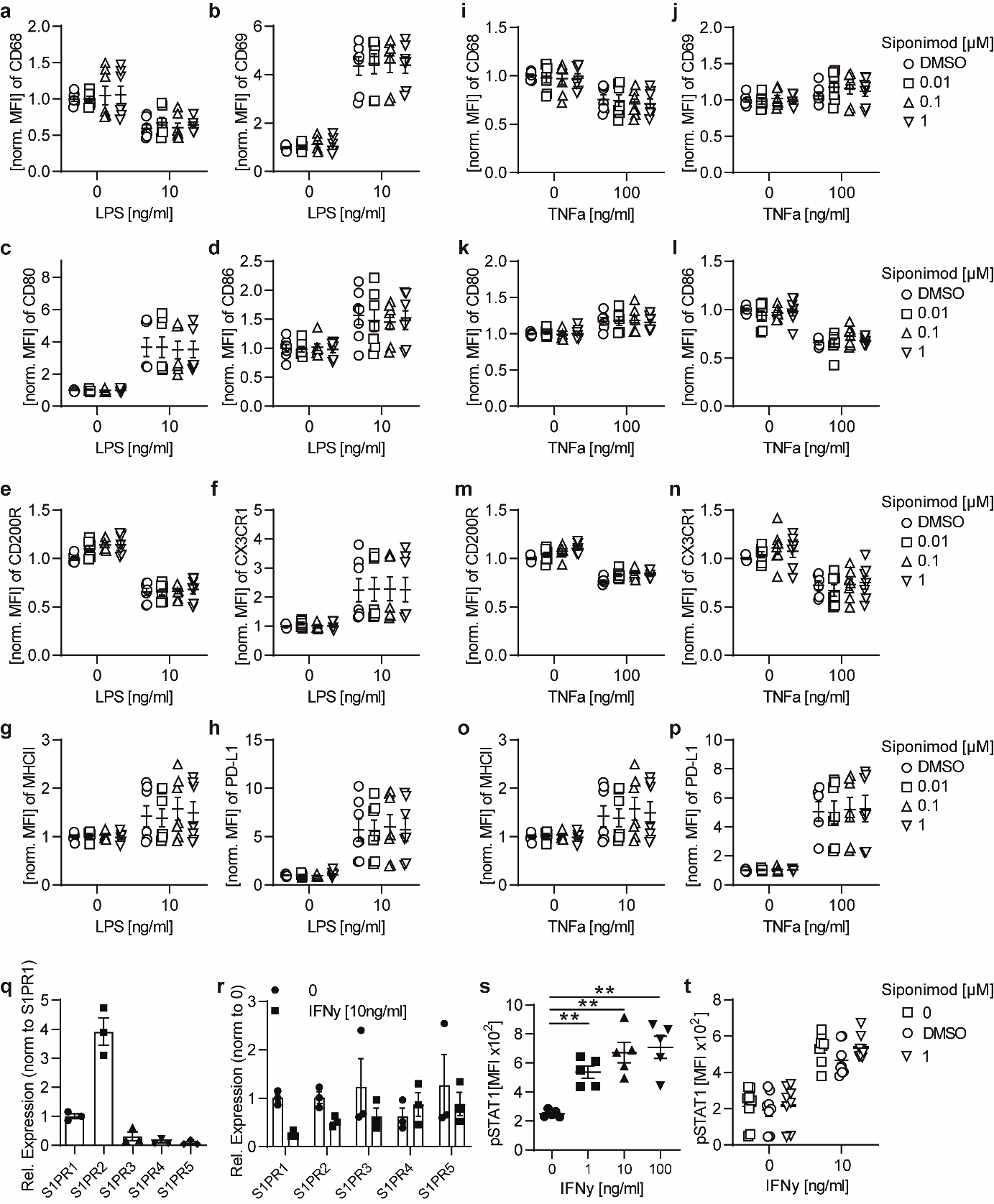
**

**Supplementary Figure 2. Siponimod pretreatment has no significant effect on LPS- and TNF-alpha-induced microglial activation in vitro.** Primary microglia were either left unstimulated or treated with indicated concentrations of siponimod or DMSO control for 24h followed by simultaneously stimulation with **a-h)** 10ng/ml LPS or **i-p)** 10ng/ml TNFa for 18h. Changes in expression of disease-associated microglial markers were analyzed by flow cytometry and normalized to DMSO control and are shown as mean fluorescence intensity, (MFI); n = 7, normalized to unstimulated control and pooled data from at least 3 independent experiments. **q-r)** Primary microglia were isolated from C57BL/6J mice and left either unstimulated of stimulated with 10ng/ml IFNγ for 18h. Cells were lysed for RNA extraction. mRNA expression of S1PR1-5 is normalized to GapDH (n = 3). **s)** Microglia were stimulated with indicated concentrations of IFNγ for 15 minutes and STAT1 phosphorylation (pSTAT1) was analyzed by flow cytometry. **t)** microglia were pretreated with 1µM siponimod or DMSO control for 24h followed by stimulation with 10 ng/ml IFNγ for 15 minutes and STAT1 phosphorylation (pSTAT1) was analyzed by flow cytometry. Mean ± standard error of the mean is indicated in all graphs. Asterisks indicate significant differences calculated using **a-p, s-t)** One-way analysis of variance corrected by Holm-Sidak. (*P ≤ 0.05, **P ≤ 0.01, ***P ≤ 0.001, ****P ≤ 0.0001).


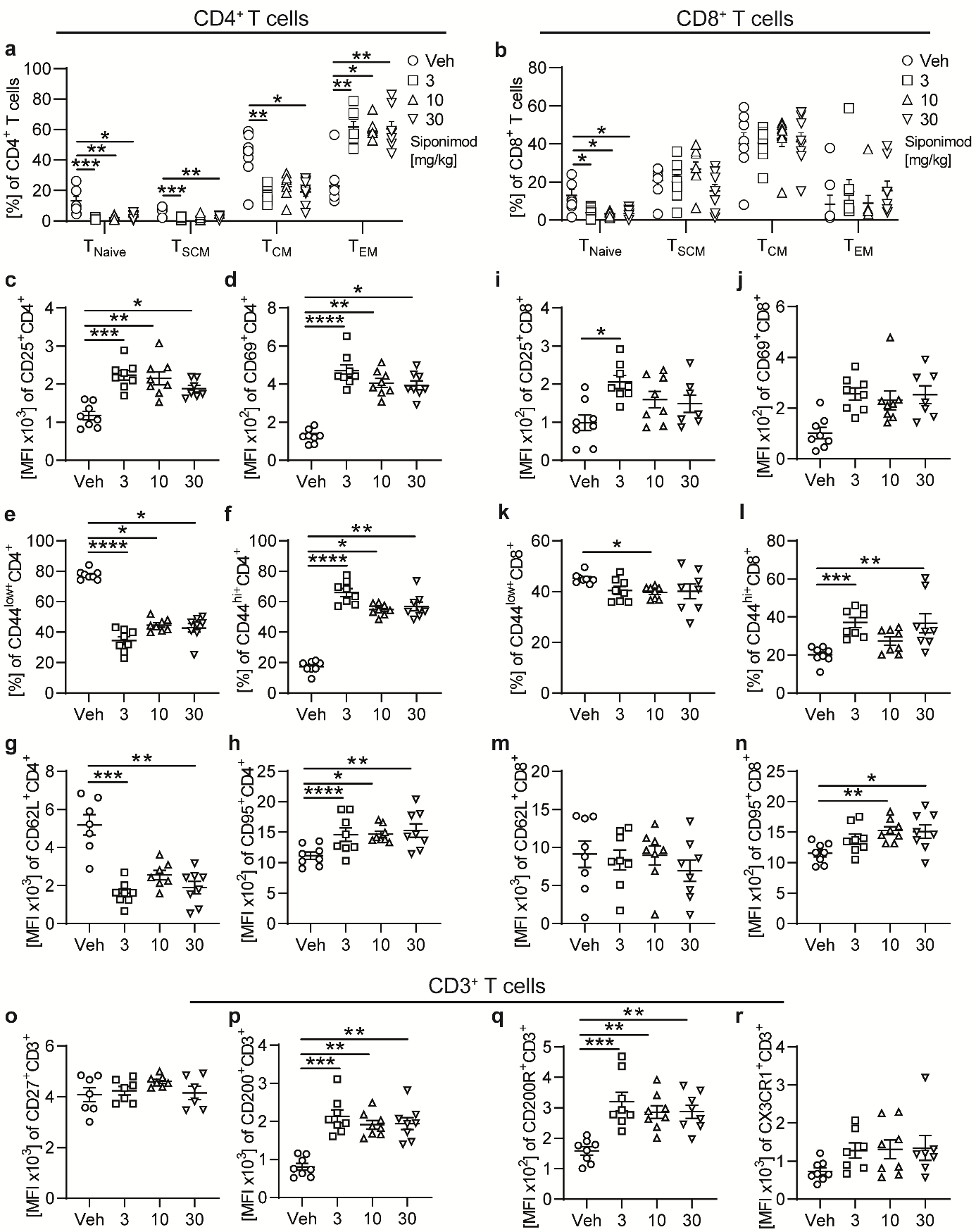


**Supplementary Figure 3. Spleen T cell subsets and activation in EAE are altered upon siponimod treatment.** EAE was induced by immunization with MOG peptide 35-55. Therapeutic treatment with food pellets loaded with siponimod at three different concentrations of 3, 10 or 30 mg per kg of food was initiated 20 days post immunization and maintained for at least 60 days. T cells were isolated from spleen. **a, b)** CD4+ and CD8+ T cell subsets (T_naive_: CD44-CD62L+CD95-; T_SCM_: CD44-CD62L+CD95+; T_CM_: CD44+CD62L+CD95+; T_EM_: CD44+CD62L-CD95+) and **c-h)** CD4+ **i-n)** CD8+ and **o-r)** CD3+ T cell activation were analyzed by flow cytometry and are shown as mean fluorescence intensity (MFI); n=8. Mean ± standard error of the mean is indicated in all graphs. Data sets are representative of three independent experiments. Asterisks indicate significant differences calculated using one-way analysis of variance Kruskal-Wallis test corrected by Dunn’s multiple comparison (*P ≤ 0.05, **P ≤ 0.01, ***P ≤ 0.001, ****P ≤ 0.0001).


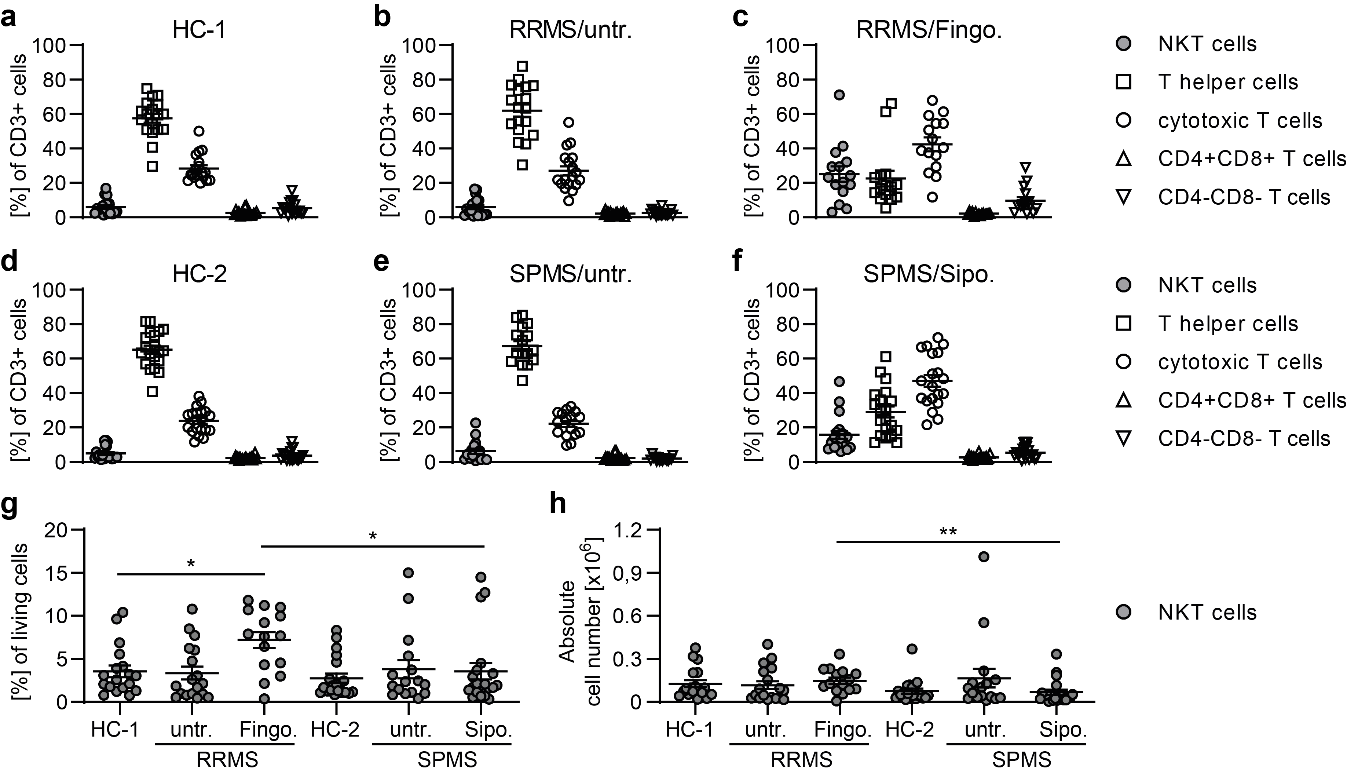


**Supplementary Figure 4. Altered composition of CD3+ blood cells under S1P receptor modulators. a-h)** Peripheral blood mononuclear cells (PBMCs) were isolated from RRMS and SPMS patients and their age- and sex-matched healthy controls (HC-1 = healthy control cohort for RRMS, n = 18; HC-2 = healthy control cohort for SPMS, n = 20) and analyzed by flow cytometry. RRMS and SPMS patients were either untreated (untr. RRMS: n = 18; untr. SPMS: n = 16) or treated with fingolimod (Fingo: n = 15) or siponimod (Sipo: n = 20), respectively. **a-f)** Frequency of blood T cell subsets representing different stages of T cell differentiation: T_N_ + T_SCM_: CD45RO-CCR7+; T_CM_: CD45RO+CCR7+; T_EM_: CD45RO+CCR7-; T_EMRA_: CD45RO-CCR7-. **g, h)** Frequency of NKT cells. Horizontal analysis of fingolimod and siponimod treatment compared to controls. Mean ± standard error of the mean is indicated in all graphs. Asterisks indicate significant differences calculated using one-way analysis of variance Kruskal-Wallis test corrected by Dunn’s multiple comparison for horizontal analysis (*P ≤ 0.05, **P ≤ 0.01, ***P ≤ 0.001, ****P ≤ 0.0001).


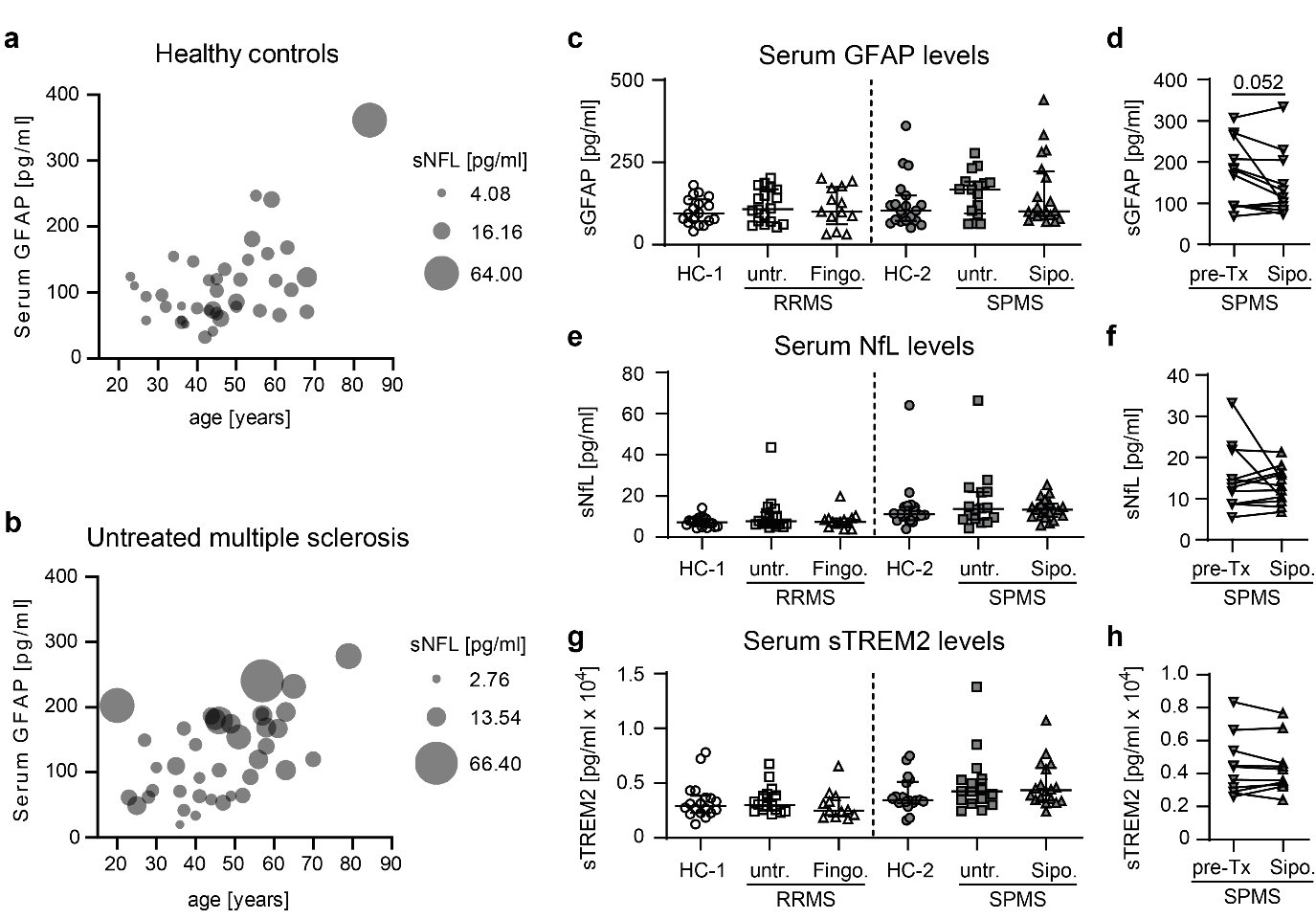


**Supplementary Figure 5. Decrease of serum GFAP levels in MS patients treated with siponimod. a-f)** Analysis of serum GFAP (sGFAP) and serum NfL (sNfL) levels in a cohort of RRMS and SPMS patients and their age- and sex-matched healthy controls (HC) using a SIMOA multiplex assay. **a-b)** Pronounced age-dependent increase of sGFAP levels in untreated multiple sclerosis (n = 39; pooled cohorts of RRMS and SPMS patients) compared to healthy controls (n = 39); each dot represents an individual subject; dot sizes correlate with individual sNFL levels. **c)** Comparison of sGFAP and sNfL levels between following cohorts: untreated (untr.) RRMS (n = 18), fingolimod treated RRMS (n = 13) and age-matched HC (HC-1, n = 18); untr. SPMS (n = 16), siponimod treated SPMS (n = 20) and age-matched HC (HC-2, n = 19). **d, f)** Longitudinal analysis with comparison of sGFAP and sNFL levels under siponimod treatment with values before treatment start (pre-Tx) in 12/20 SPMS. **g, h)** Serum levels of microglial activity marker sTREM2 were determined using a SIMOA assay: **g)** Horizontal analysis of study cohorts: untr. RRMS (n = 16), fingolimod-treated RRMS (n = 12) and age-matched HC (HC-1, n = 18); untr. SPMS (n = 16), siponimod-treated SPMS (n = 18) and age-matched HC (HC-2, n = 18) **h)** Longitudinal analysis of sTREM2 levels in SPMS under siponimod treatment versus pre-Tx (n = 10). All graphs show median values and quartiles. Normal distribution was tested with a Shapiro-Wilk normality test. In the case of normal distribution, an ordinary one-way ANOVA test corrected with a Holm-Sidak’s multiple comparisons test was applied for horizontal comparison of different treatment groups; a two-tailed Paired t test was used for longitudinal analysis. When normal distribution was not confirmed, a one-way analysis of variance Kruskal-Wallis test corrected by Dunn’s multiple comparison was performed for horizontal analysis and a Wilcoxon matched-pairs signed rank test for longitudinal analysis. Asterisks indicate significant differences (*P ≤ 0.05, **P ≤ 0.01, ***P ≤ 0.001, ****P ≤ 0.0001).


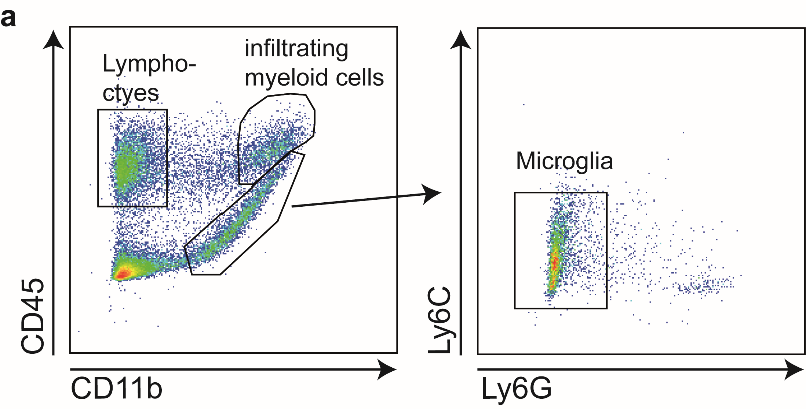


**Supplementary Figure 6. Gating strategy to distinguish infiltrating myeloid cells from CNS-resident microglia**. Gating strategy of microglia isolated from the CNS (CD11b^+^CD45^int^Ly6C^-^Ly6G^-^).

**Supplementary Table 1. Pretreatment in the siponimod treatment cohort**

| **Horizontal**  **analysis** | **Pretreatment with disease-modifying therapies**  (treatment-free interval prior siponimod) | | |
| --- | --- | --- | --- |
| patient 1 | interferon beta-1a  (60 months) |  |  |
| patient 2 | mitoxantrone  (60 months) |  |  |
| patient 3 | no prior treatment  (not applicable) |  |  |
| patient 4 | interferon beta-1a  (no treatment-free interval) | | |
| patient 5 | interferon beta-1a  (48 months) |  |  |
| patient 6 | ocrelizumab  (22 months) |  |  |
| patient 7 | azathioprine  (data missing) |  |  |
| **Horizontal and**  **longitudinal analysis** | **Pretreatment with disease-modifying therapies** (treatment-free interval prior siponimod) | | |
| patient 8 | no prior treatment  (not applicable) |  |  |
| patient 9 | interferon beta-1b  (2 months) |  |  |
| patient 10 | no prior treatment  (not applicable) |  |  |
| patient 11 | no prior treatment  (not applicable) |  |  |
| patient 12 | dimethyl fumarate  (2 months) |  |  |
| patient 13 | no prior treatment  (not applicable) |  |  |
| patient 14 | glatiramer acetate  (no treatment-free interval) | | |
| patient 15 | dimethyl fumarate  (1.5 months) |  |  |
| patient 16 | mitoxantrone  (31 months) |  |  |
| patient 17 | no prior treatment  (not applicable) |  |  |
| patient 18 | glatiramer acetate  (3 months) |  |  |
| patient 19 | dimethyl fumarate  (16 months) |  |  |
| patient 20 | dimethyl fumarate  (1.5 months) |  |  |

**Supplementary Table 2. Information on antibodies for immunohistochemistry specific for murine antigens**

| **Target antigen** | **Vendor** | **Cat. number** | **RRID number** | **Dilution** |
| --- | --- | --- | --- | --- |
| B220 (CD45R) | BD Biosciences | 557390 | AB_396673 | 1:200 |
| CD3 | DCS Innovative Diagnostik-Systeme | CI597C01 | n/a | 1:50 |
| Iba1 | Fujifilm | 019-19741 | AB_839504 | 1:500 |
| MAC-3 | BD Biosciences | 553322 | AB_394780 | 1:200 |
| Olig2 | IBL - America | 18953 | AB_2267671 | 1:300 |

**Supplementary Table 3. Information on antibodies for flow cytometry specific for human antigens**

| **Target antigen** | **Vendor** | **Cat. number** | **RRID number** | **Dilution** |
| --- | --- | --- | --- | --- |
| CD3 | BD Biosciences | 563546 | AB_2744387 | 1:100 |
| CD3 | BioLegend | 300448 | AB_2563468 | 1:100 |
| CD4 | BioLegend | 300512 | AB_314080 | 1:100 |
| CD8 | BioLegend | 300933 | AB_2814114 | 1:100 |
| CD8 | BD Biosciences | 560662 | AB_1727513 | 1:100 |
| CD14 | BioLegend | 301808 | AB_314190 | 1:100 |
| CD14 | BD Biosciences | 565283 | AB_2739154 | 1:100 |
| CD19 | BioLegend | 302230 | AB_2073119 | 1:100 |
| CD25 | BioLegend | 302629 | AB_10896914 | 1:100 |
| CD27 | BioLegend | 356417 | AB_2562598 | 1:100 |
| CD45RA | BD Biosciences | 563031 | AB_2722499 | 1:100 |
| CD45RO | BioLegend | 560899 | AB_10563936 | 1:100 |
| CD56 | BD Biosciences | 562794 | AB_2737799 | 1:50 |
| CD62L | BioLegend | 304827 | AB_10896429 | 1:100 |
| CD69 | BioLegend | 310932 | AB_2563696 | 1:100 |
| CD95 | BioLegend | 305608 | AB_314546 | 1:100 |
| CD127 | BD Biosciences | 557938 | AB_2296056 | 1:100 |
| CD197 (CCR7) | BD Biosciences | 562381 | AB_11153301 | 1:100 |
| CX3CR1 | BioLegend | 355703 | AB_2561680 | 1:100 |
| CD16/CD32 | BioLegend | 422302 | AB_2818986 | 1:100 |

**Supplementary Table 4. Information on antibodies for flow cytometry specific for murine antigens**

| **Target antigen** | **Vendor** | **Cat. number** | **RRID number** | **Dilution** |
| --- | --- | --- | --- | --- |
| CD3 | BD Biosciences | 563565 | AB_2738278 | 1:100 |
| CD4 | BioLegend | 100559 | AB_2562608 | 1:100 |
| CD8 | BioLegend | 100706 | AB_312745 | 1:100 |
| CD11b | BioLegend | 101206 | AB_312789 | 1:100 |
| CD11c | BioLegend | 117348 | AB_2563655 | 1:100 |
| CD19 | BioLegend | 115546 | AB_2562137 | 1:100 |
| CD20 | BioLegend | 150404 | AB_2565974 | 1:200 |
| CD25 | BioLegend | 102008 | AB_312857 | 1:100 |
| CD27 | BioLegend | 124211 | AB_1236460 | 1:100 |
| CD44 | BioLegend | 103012 | AB_312963 | 1:100 |
| CD45 | BioLegend | 103132 | AB_893340 | 1:100 |
| CD45 | BD Biosciences | 564279 | AB_2651134 | 1:100 |
| CD62L | BioLegend | 104418 | AB_313103 | 1:100 |
| CD68 | BioLegend | 137008 | AB_10575300 | 1:100 |
| CD69 | BioLegend | 104522 | AB_2260065 | 1:100 |
| CD69 | BioLegend | 104536 | AB_2565583 | 1:100 |
| CD80 | BD Biosciences | 562504 | AB_2737630 | 1:100 |
| CD86 | BioLegend | 105032 | AB_2650895 | 1:100 |
| CD95 | BD Biosciences | 562633 | AB_2737690 | 1:100 |
| CD200 | BioLegend | 123820 | AB_2832446 | 1:100 |
| CD200R | BioLegend | 123908 | AB_2074080 | 1:100 |
| CX3CR1 | BioLegend | 149023 | AB_2565706 | 1:100 |
| Ly6C | BioLegend | 128036 | AB_2562353 | 1:100 |
| Ly6C | BioLegend | 128032 | AB_2562178 | 1:100 |
| Ly6C | BioLegend | 128044 | AB_2566577 | 1:100 |
| Ly6C | BioLegend | 128016 | AB_1732076 | 1:100 |
| Ly6G | BioLegend | 127616 | AB_1877271 | 1:100 |
| Ly6G | BioLegend | 127614 | AB_2227348 | 1:100 |
| MHCII | BioLegend | 116408 | AB_313727 | 1:100 |
| PD-L1 | eBioscience | 12-5982-82 | AB_466089 | 1:100 |
| Stat1 (pY701) | BD Biosciences | 612597 | AB_399880 | 20µl/test |
| CD16/CD32 | BioLegend | 101320 | AB_1574975 | 1:100 |
